# Design, Synthesis, and Bioactivity of Novel Quinazolinone Scaffolds Containing Pyrazole Carbamide Derivatives as Antifungal Agents

**DOI:** 10.3390/cimb44110380

**Published:** 2022-11-12

**Authors:** Zhiwei Lei, Jianmei Yao, Huifang Liu, Xianjin Bai, Xingsi Gao, Qiuyuan Pan, Wen Yang

**Affiliations:** 1 Key Laboratory of Green Pesticide and Agricultural Bioengineering, Ministry of Education, Guizhou University, Huaxi District, Guiyang 550025, China; 2 Tea Research Institute, Guizhou Academy of Agricultural Sciences, Huaxi District, Guiyang 550006, China; 3 School of Biological Sciences, Guizhou Education University, Wudang District, Guiyang 550000, China

**Keywords:** fungicidal activity, quinazolinone, pyrazolecarbamide, structure-activity relationship

## Abstract

In this study, 32 novel quinazolinone-scaffold-containing pyrazole carbamide derivatives were designed and synthesized in a search for a novel fungicide against *Rhizoctonia solani*. Single-crystal X-ray diffraction of 3-(difluoromethyl)-*N*-(2-((6,7-difluoro-4-oxoquinazolin-3(4H)-yl)methyl)phenyl)-1-methyl-1H-pyrazole-4-carboxamide (**6a_11_**) confirmed the structure of the target compounds. The in vitro antifungal activity of the target compounds against *R. solani* was evaluated at 100 µg/mL. The structure–activity relationship analysis results revealed that antifungal activity was highest when the substitution activity was at position 6. Moreover, the position and number of chlorine atoms directly affected the antifungal activity. Further in vitro bioassays revealed that **6a_16_** (EC_50_ = 9.06 mg/L) had excellent antifungal activity against *R. solani* that was higher than that of the commercial fungicide fluconazole (EC_50_ = 12.29 mg/L) but lower than that of bixafen (EC_50_ = 0.34 mg/L). Scanning electron microscopy), 7.33 (SEM) revealed that *N*-(2-((6,8-dichloro-4-oxoquinazolin-3(4H)-yl)methyl)phenyl)-3-(difluoromethyl)-1-methyl-1H-pyrazole-4-carboxamide (**6a_16_**) also affected the mycelial morphology. The findings revealed that molecular hybridization was an effective tool for designing antifungal candidates. Meanwhile, pyrazolecarbamide derivatives bearing a quinazolinone fragment exhibited potential antifungal activity against *R. solani*.

## 1. Introduction

*Rhizoctonia solani* Kunh can infect the leaves and roots of various crops [1,2], causing significant yield loss. Specifically, *R. solani* causes sheath blight in rice, leading up to 40% yield loss [3,4]. Currently, chemical pesticides remain the most effective strategy for controlling rice sheath blight, because of the lack of rice sheath blight-resistant varieties. However, the abuse of chemical pesticides leads to pesticide resistance by pathogenic fungi. Therefore, new pesticide formulations that are more effective and more friendly to the environment need to be continuously developed.

Heterocyclic compounds are the largest class of organic compounds and play a significant role in the development of pesticides, irrespective of whether they are natural or synthetic [5,6]. New super-efficient pesticides, most of which contain heterocyclic compounds, are continuously being developed. Nitrogen-containing heterocyclic compounds, such as pyrazole and quinazolinone, have been found to have good anti-fungal activity.

**Figure 1:**
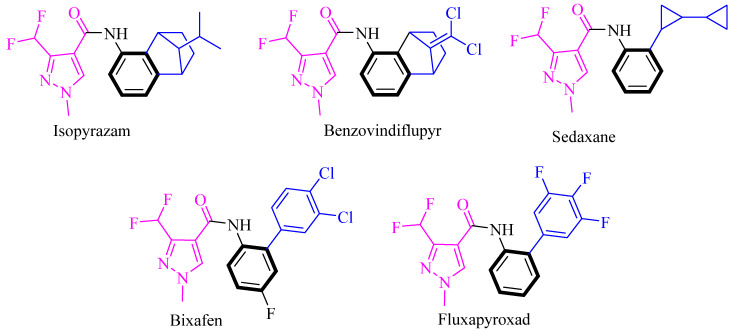
Representative fungicides of pyrazolecarboxamides.

Notably, pyrazole amides have become a hot spot in fungicide research because of their unique mechanism of action, safety, and efficiency [7]. Pesticide companies have developed pyrazole amide fungicides, such as isopyrazam [8], benzovindiflupyr [9], sedaxane [10], bixafen [11], and fluxapyroxad [12] (Figure 1), whose common feature is their connection by benzene rings as bridges.

**Figure 2:**
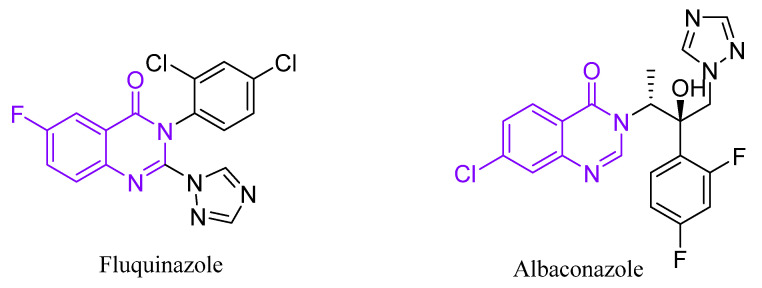
Representative fungicides of quinazolinone.

Quinazolinone is a benzopyrimidine heterocyclic compound that is the backbone structure of various alkaloids and drugs [13,14]. It has been widely used in the fields of medicine and pesticides because of its various excellent properties, such as its antifungal [15,16], antibacterial [17], antitumor [18], and antiviral activities [19,20]. For example, quinazolinone derivatives have been successfully developed into a commercial fungicide, fluconazole, which is used to control various fungal diseases caused by basidiomycetes, deuteromycetes, and discomycetes [21,22]. Albaconazole [23], which contains quinazolin-4-one, also exhibits a broad-spectrum antifungal activity (Figure 2).

**Figure 3:**
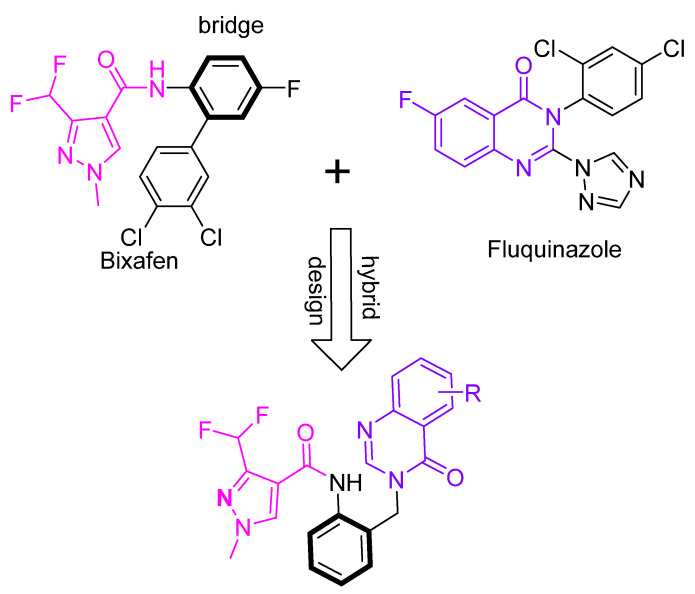
The design strategy of the target compounds.

A molecular hybrid is a combination of two or more independently acting pharmacophores that are covalently linked [24]. It can be achieved by linking or by framework integration of two or more molecules to form one molecule with increased pharmacological activity. In this study, a quinazolinone structural unit and a pyrazole-containing active fragment were combined into one molecular structure. The two were connected using a benzene ring and used to design and synthesize a series of quinazolinone-containing pyrazole carboxamide derivatives (Figure 3). The molecular structure of the hybrid molecule was determined using ^1^H NMR, ^13^C NMR, and HRMS, followed by a preliminary screening of its antifungal activity in vitro. Subsequent physiological assays of compounds with high antifungal activity against *R. solani*, which causes sheath blight in rice, were further conducted.

## 2. Materials and Methods

### 2.1. Chemistry

#### 2.1.1. Instruments and Chemicals

^1^H and ^13^C NMR spectra were recorded in DMS*O*-*d_6_* using 400 and 100 MHz spectrophotometers (Bruker BioSpin GmbH, Rheinstetten, Germany), respectively, while high-resolution mass spectrometry (HRMS) was performed using Thermo Scientific Q Exactive (Thermo Fisher Scientific, Waltham, MA, USA). The X-ray crystallographic data were collected and processed using a D8 Quest X-ray diffractometer (Bruker BioSpin GmbH, Rheinstetten, German). All solvents were distilled and dried using standard methods before use.

#### 2.1.2. Synthesis of Intermediate **3**

**Scheme 1:**
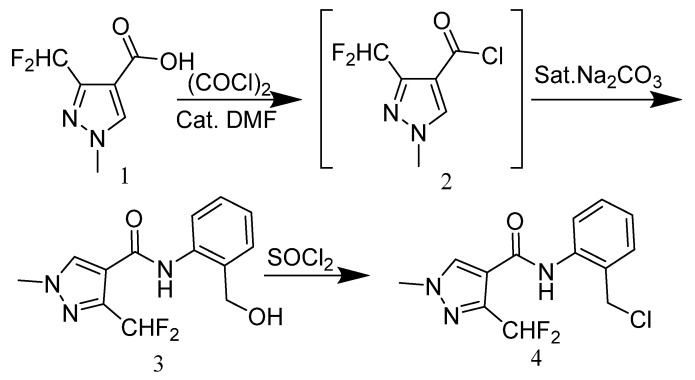
Synthesis of key intermediates of pyrazole carboxamide.

*O*-aminobenzyl alcohol (14.10 g, 100 mmol) was first added to 300 mL of saturated sodium bicarbonate solution, followed by dropwise addition of 3-(difluoromethyl)-1-methyl-1H-pyrazole-4-carbonyl chloride [25] (**3**, 23.28 g, 120 mmol) at room temperature. A huge amount of white solid was produced and filtered. The filtered cake was then recrystallized from ethanol to obtain a white product 3-(difluoromethyl)-*N*-(2-(hydroxymethyl)phenyl)-1-methyl-1H-pyrazole-4-carboxamide (**3**, Scheme 1). Intermediate **3**, white solid, yield 90%, m.p. 199.7–200.6 °C. ^1^H NMR (400 MHz, DMS*O*-*d_6_*) *δ* 9.74 (s, 1H, *NH*), 8.39 (s, 1H, pyrazol-*H5*), 7.57 (d, *J* = 7.5 Hz, 1H, Ar-*H5*), 7.45 (dd, *J* = 7.6, 1.7 Hz, 1H, Ar-*H2*), 7.32 (t, *J* = 54.3 Hz, 1H, CF_2_*H*), 7.28 (td, *J* = 7.6, 1.7 Hz, 1H, Ar-*H3*), 7.20 (td, *J* = 7.5, 1.4 Hz, 1H, Ar-*H4*), 5.43 (s, 1H, O*H*), 4.56 (s, 2H, *CH_2_*), 3.97 (s, 3H, *CH_3_*). ^13^C NMR (100 MHz, DMS*O*-*d_6_*) *δ* 159.79, 144.88 (t, *J* = 23.3 Hz), 135.36, 135.08, 132.68, 127.38, 127.11, 125.18, 124.44, 116.08 (t, *J* = 3.6 Hz), 109.72 (t, *J* = 234.6 Hz), 60.41, 39.44. HRMS (ESI): calculated for C_13_H_13_F_2_N_3_O_2_ [M+Na]^+^: 304.08680, found: 304.08713.

#### 2.1.3. Synthesis of Intermediate **4**

The intermediate (**3**, 1 mmol) was dissolved in DMF (5 mL), followed by the addition of triethylamine (1 mmol) and thionyl chloride (3 mmol) at room temperature and stirring for 30 min. The reaction solution was then poured into a saturated sodium bicarbonate solution (100 mL) and extracted thrice using ethyl acetate. The extract was dried over sodium sulfate, filtered, concentrated under reduced pressure, and then subjected to column chromatography (PE/EA = 3/2) to obtain a white product *N*-(2-(chloromethyl)phenyl)-3-(difluoromethyl)-1-methyl-1H-pyrazole-4-carboxamide (**4**, Scheme 1). Intermediate **4**, white needle, yield 75%. m.p. 119.9–121.1 °C. ^1^H NMR (400 MHz, DMS*O*-*d_6_*) *δ* 9.85 (s, 1H, N*H*), 8.49 (s, 1H, pyrazol-*H5*), 7.52 (dd, *J* = 7.6, 1.5 Hz, 1H, Ar-*H5*), 7.47–7.36 (m, 2H, Ar-*H2*, *H3*), 7.33–7.24 (m, 2H, CF_2_*H*, Ar-*H4*), 4.82 (s, 2H, *CH_2_*), 3.98 (s, 3H, *CH_3_*). ^13^C NMR (100 MHz, DMS*O*-*d_6_*) *δ* 160.25, 145.09 (t, *J* = 23.4 Hz), 135.77, 133.16, 132.60, 130.38, 129.03, 127.05, 126.29, 115.84, 115.82 (t, *J* = 3.7 Hz), 109.71 (t, *J* = 234.7 Hz), 43.27, 39.45. HRMS (ESI): calculated for C_13_H_12_ClF_2_N_3_O [M+Na]^+^: 322.05292, found: 322.05263.

#### 2.1.4. General Procedure for the Preparation of Quinazolin-4-Ones [26] (**5a_1_-a_32_**)

**Scheme 2:**
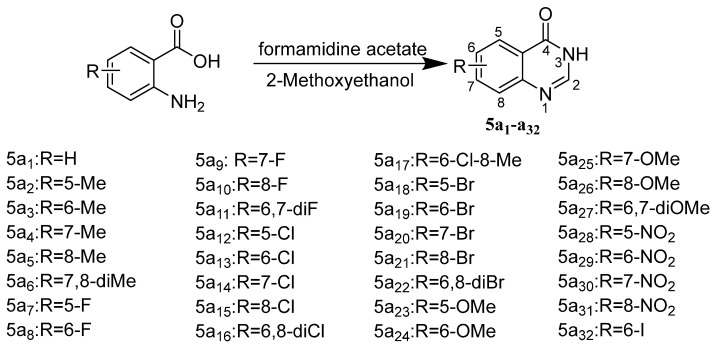
Synthesis of key intermediate quinazolinone.

Anthranilic acid or its substituted derivatives (10 mmol) were mixed with formamidine acetate (20 mmol) in ethylene glycol monomethyl ether and the reaction mixture was subsequently stirred at 95–130 °C. The mixture was then poured into cold water, thereby precipitating a large amount of solid. The crude product was subsequently obtained by filtration and was dissolved in hot 10% NaOH solution, heated for 5–6 min with charcoal and filtered, and the clear solution was subsequently neutralized (pH = 7) using 1N HCl. The precipitated crystals were filtered out, washed with cooled water, and dried to obtain the quinazolin-4-ones (Scheme 2, Supplementary Materials).

#### 2.1.5. General Procedure for the Preparation of the Target Compounds **6a_1_-a_32_**

**Scheme 3:**
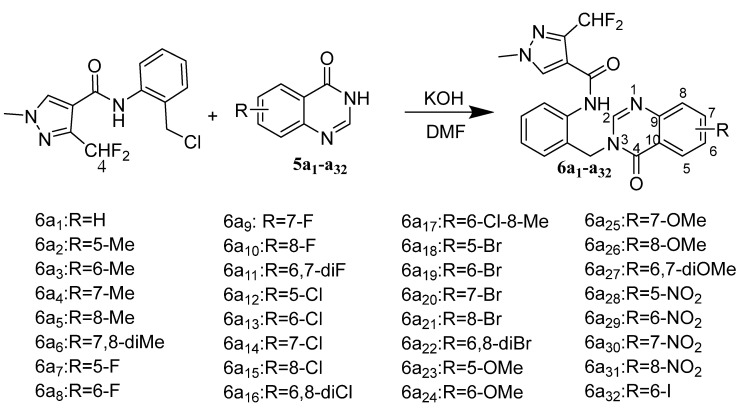
Synthesis of the target compounds **6a_1_-a_32_**.

Substituted quinazolinone (1 mmol, **5a_1_-a_32_**) was added to 10 mL DMF and stirred to dissolve. KOH (59 mg, 1.05) was added and left to react for 40 min, after which **4** (299 mg, 1 mmol) was added. The reaction was carried out at room temperature for 1–3 h. The reaction was detected by TLC, followed by the addition and stirring of 20 mL of saturated ammonium chloride solution for 5 min. It was then extracted with dichloromethane (20 mL × 3), dried over anhydrous sodium sulfate, filtered, concentrated, and purified (Scheme 3).

3-(difluoromethyl)-1-methyl-N-(2-((4-oxoquinazolin-3(4H)-yl)methyl)phenyl)-1H-pyrazole-4-carboxamide (**6a_1_**), pale white powder, yield 83%, m.p. 172.8–174.7 °C. 1H NMR (400 MHz, DMSO-*d_6_*) δ 10.21 (s, 1H, *NH*), 8.54 (s, 1H, *pyrazol-H5*), 8.50 (s, 1H, *H2*), 8.17 (dd, *J* = 8.0, 1.5 Hz, 1H, *H5*), 7.84 (ddd, *J* = 8.4, 7.1, 1.5 Hz, 1H, *H7*), 7.70 (dd, *J* = 8.1, 1.1 Hz, 1H, *H8*), 7.55 (ddd, *J* = 8.2, 7.1, 1.2 Hz, 1H, *H6*), 7.49 (dd, *J* = 8.0, 1.3 Hz, 1H, Ar-*H2*), 7.35 (td, *J* = 7.6, 1.8 Hz, 1H, Ar-*H3*), 7.31 (t, *J* = 54.3 Hz, 1H, CF_2_*H*), 7.28 (dd, *J* = 7.8, 1.8 Hz, 1H, Ar-*H5*), 7.23 (dd, *J* = 7.6, 1.4 Hz, 1H, Ar-*H4*), 5.23 (s, 2H,*CH_2_*), 4.01 (s, 3H, *pyrazol-CH_3_*). ^13^C NMR (100 MHz, DMSO-*d_6_*) δ 160.63, 160.22, 147.93, 147.90, 145.18 (t, *J* = 23.1 Hz), 135.36, 134.59, 132.90, 131.20, 128.48, 128.22, 127.28, 127.25, 126.40, 126.20, 126.17, 121.40, 115.91 (t, *J* = 3.7 Hz), 109.64 (t, *J* = 234.6 Hz), 59.77, 45.87. HRMS (ESI): calculated for C_21_H_17_F_2_N_5_O_2_ [M+Na]^+^: 432.12425, found: 432.12577.

3-(difluoromethyl)-1-methyl-*N*-(2-((5-methyl-4-oxoquinazolin-3(4H)-yl)methyl)phenyl)-1H-pyrazole-4-carboxamide (**6a_2_**), white powder, yield 71%, m.p. 223.1–224.2 °C. ^1^H NMR (400 MHz, DMS*O*-*d_6_*) *δ* 10.22 (s, 1H,*NH*), 8.55 (s, 1H, *pyrazol-H5*), 8.44 (s, 1H, *H2*), 7.65 (t, *J* = 7.7 Hz, 1H, *H7*), 7.53–7.47 (m, 2H, *H8, Ar-H2*), 7.35 (td, *J* = 7.6, 1.7 Hz, 1H, Ar-*H3*), 7.32–7.26 (m, 3H, CF_2_*H*, *H6*, *Ar-H5*), 7.22 (td, *J* = 7.5, 1.3 Hz, 1H, *Ar-H4*), 5.17 (s, 2H, *CH_2_*), 3.99 (s, 3H, *pyrazol-CH_3_*), 2.73 (s, 3H, *5-CH_3_*). ^13^C NMR (100 MHz, DMS*O*-*d_6_*) *δ* 161.20, 160.14, 149.54, 147.58, 145.15 (t, *J* = 23.1 Hz), 140.22, 135.44, 133.67, 132.93, 131.23, 129.60, 128.62, 128.20, 126.15, 126.07, 125.49, 119.85, 115.94 (t, *J* = 3.7 Hz), 109.61 (t, *J* =234.7 Hz), 45.60, 39.48, 22.73. HRMS (ESI): calculated for C_22_H_19_F_2_N_5_O_2_ [M+Na]^+^: 446.13990, found: 446.13964.

3-(difluoromethyl)-1-methyl-*N*-(2-((6-methyl-4-oxoquinazolin-3(4H)-yl)methyl)phenyl)-1H-pyrazole-4-carboxamide (**6a_3_**), white powder, yield 72%, m.p. 258.8–260.1 °C. ^1^H NMR (400 MHz, DMS*O*-*d_6_*) *δ* 10.21 (s, 1H, *NH*), 8.54 (s, 1H, *pyrazol-H5*), 8.43 (s, 1H, *H2*), 7.99–7.90 (m, 1H, *H5*), 7.66 (dd, *J* = 8.4, 2.1 Hz, 1H, *H7*), 7.60 (d, *J* = 8.3 Hz, 1H, *H8*), 7.49 (dd, *J* = 8.0, 1.2 Hz, 1H, *Ar-H2*), 7.38–7.31 (m, 1H, Ar-*H3*), 7.31 (t, *J* = 54.3 Hz, 1H, CF_2_*H*), 7.25 (dd, *J* = 7.8, 2.0 Hz, 1H, *Ar-H5*), 7.23–7.18 (m, 1H, *Ar-H4*), 5.22 (s, 2H, *CH_2_*), 4.01 (s, 3H, *pyrazol-CH_3_*), 2.44 (s, 3H, *6-CH_3_*). ^13^C NMR (100 MHz, DMS*O*-*d_6_*) *δ* 160.54, 160.16, 147.06, 145.96, 145.15 (t, *J* = 23.1 Hz), 137.11, 135.88, 135.31, 132.87, 131.20, 128.37, 128.17, 127.12, 126.36, 126.14, 125.48, 121.12, 115.89 (t, *J* = 3.7 Hz), 109.62 (t, *J* = 234.7 Hz), 45.73, 39.52, 20.82. HRMS (ESI): calculated for C_22_H_19_F_2_N_5_O_2_ [M+Na]^+^: 446.13975, found: 446.13990.

3-(difluoromethyl)-1-methyl-*N*-(2-((7-methyl-4-oxoquinazolin-3(4H)-yl)methyl)phenyl)-1H-pyrazole-4-carboxamide (**6a_4_**), white powder, yield 61%, m.p. 246.5–246.8 °C. ^1^H NMR (400 MHz, DMS*O*-*d_6_*) *δ* 10.24 (s, 1H, *NH*), 8.55 (s, 1H, *pyrazol-H5*), 8.48 (s, 1H, *H2*), 8.06 (d, *J* = 8.1 Hz, 1H, *H5*), 7.54–7.47 (m, 2H, *H6,Ar-H2*), 7.38 (dd, *J* = 8.4, 1.8 Hz, 1H, *H8*), 7.36–7.32 (m, 1H, Ar-*H3*), 7.31 (t, *J* = 54.3 Hz, 1H, CF_2_*H*), 7.27 (dd, *J* = 7.8, 1.7 Hz, 1H, *Ar-H5*), 7.21 (td, *J* = 7.5, 1.3 Hz, 1H, *Ar-H4*), 5.21 (s, 2H, *CH_2_*), 4.01 (s, 3H, *pyrazol-CH_3_*), 2.46 (s, 3H, *CH_3_*). ^13^C NMR (100 MHz, DMS*O*-*d_6_*) *δ* 160.54, 160.16, 148.08, 147.94, 145.25, 145.16 (t, *J* = 23.3 Hz), 135.36, 132.87, 131.10, 128.70, 128.50, 128.21, 126.85, 126.27, 126.09, 126.06, 118.97, 115.91 (t, *J* = 3.7 Hz), 109.63 (t, *J* = 234.5 Hz), 45.71, 39.52, 21.31. HRMS (ESI): calculated for C_22_H_19_F_2_N_5_O_2_ [M+Na]^+^: 446.13990, found: 446.13975.

3-(difluoromethyl)-1-methyl-*N*-(2-((8-methyl-4-oxoquinazolin-3(4H)-yl)methyl)phenyl)-1H-pyrazole-4-carboxamide (**6a_5_**), white powder, yield 76%, m.p. 215.8–216.6 °C. ^1^H NMR (400 MHz, DMS*O*-*d_6_*) *δ* 10.22 (s, 1H, *NH*), 8.54 (s, 1H, *pyrazol-H5*), 8.53 (s, 1H, *H2*), 8.01 (ddd, *J* = 8.0, 1.6, 0.7 Hz, 1H, *H5*), 7.70 (ddd, *J* = 7.3, 1.6, 0.9 Hz, 1H, *H7*), 7.49 (dd, *J* = 8.0, 1.3 Hz, 1H, *H6*), 7.47–7.42 (m, 1H, Ar-*H2*), 7.38–7.32 (m, 1H, Ar-*H3*), 7.31 (t, *J* = 54.16 Hz, 1H, CF_2_*H*), 7.26 (dd, *J* = 7.8, 1.9 Hz, 1H, *Ar-H5*), 7.24–7.18 (m, 1H, *Ar-H4*), 5.21 (s, 2H, *CH_2_*), 4.01 (s, 3H, *pyrazol-CH_3_*), 2.54 (s, 3H, 8-*CH_3_*). ^13^C NMR (100 MHz, DMS*O*-*d_6_*) *δ* 160.85, 160.19, 146.95, 146.41, 145.15 (t, *J* = 23.2 Hz), 135.52, 135.31, 134.94, 132.89, 131.16, 128.39, 128.19, 126.82, 126.32, 126.15, 123.87, 121.33, 115.89 (t, *J* = 3.4 Hz), 109.63 (t, *J* = 234.4 Hz), 45.82, 39.31, 17.03. HRMS (ESI): calculated for C_22_H_19_F_2_N_5_O_2_ [M+Na]^+^: 446.13990, found: 446.13975.

3-(difluoromethyl)-*N*-(2-((7,8-dimethyl-4-oxoquinazolin-3(4H)-yl)methyl)phenyl)-1-methyl-1H-pyrazole-4-carboxamide (**6a_6_**), white powder, yield 61%, m.p. 269.0–270.4 °C. ^1^H NMR (400 MHz, DMS*O*-*d_6_*) *δ* 10.25 (s, 1H, *NH*), 8.54 (s, 1H, *pyrazol-H5*), 8.51 (s, 1H, *H2*), 7.93 (d, *J* = 8.1 Hz, 1H, *H5*), 7.50 (dd, *J* = 8.0, 1.3 Hz, 1H, *H6*), 7.37 (d, *J* = 8.2 Hz, 1H, *Ar-H2*), 7.33 (dd, *J* = 7.9, 1.8 Hz, 1H, Ar-*H3*), 7.30 (t, *J* = 54.1 Hz, 1H, CF_2_*H*), 7.26 (dd, *J* = 7.8, 1.7 Hz, 1H, *Ar-H5*), 7.24–7.18 (m, 1H, *Ar-H4*), 5.20 (s, 2H, *CH_2_*), 4.01 (s, 3H, *pyrazol-CH_3_*), 2.48 (s, 3H, 8-*CH_3_*), 2.40 (s, 3H, 7-*CH_3_*). ^13^C NMR (100 MHz, DMS*O*-*d_6_*) *δ* 160.92, 160.15, 146.71, 146.12, 145.15 (t, *J* = 23.5 Hz), 143.28, 135.33, 133.50, 132.87, 131.06, 128.99, 128.44, 128.19, 126.20, 126.08, 123.05, 119.24, 115.92 (t, *J* = 3.6 Hz), 109.63 (t, *J* = 234.7 Hz), 45.62, 39.52, 20.46, 12.77. HRMS (ESI): calculated for C_23_H_21_F_2_N_5_O_2_ [M+Na]^+^: 460.15555, found: 460.15515.

3-(difluoromethyl)-*N*-(2-((5-fluoro-4-oxoquinazolin-3(4H)-yl)methyl)phenyl)-1-methyl-1H-pyrazole-4-carboxamide (**6a_7_**), white powder, yield 66%, m.p. 243.1–244.5 °C. ^1^H NMR (400 MHz, DMS*O*-*d_6_*) *δ* 10.11 (s, 1H, *NH*), 8.52 (s, 1H, *pyrazol-H5*), 8.45 (s, 1H, *H2*), 7.81 (td, *J* = 8.2, 5.5 Hz, 1H, *H7*), 7.50 (dd, *J* = 8.3, 1.0 Hz, 1H, *H8*), 7.44 (dd, *J* = 8.0, 1.3 Hz, 1H, *H6*), 7.38–7.29 (m, 2H, *Ar-H2*, *Ar-H3*), 7.28 (t, *J* = 54.1 Hz, 1H, CF_2_*H*), 7.27–7.20 (m, 2H, *Ar-H4*, *Ar-H5*), 5.17 (s, 2H, *CH_2_*), 3.99 (s, 3H, *pyrazol-CH_3_*). ^13^C NMR (100 MHz, DMS*O*-*d_6_*) 160.34 (d, *J* = 263.6 Hz, 1H), 160.26, 159.03, 157.54 (d, *J* = 3.8 Hz, 1H), 157.52, 150.09, 148.88, 145.13 (t, *J* = 23.0 Hz), 135.33, 135.24 (d, *J* = 10.6 Hz, 1H), 132.22 (d, *J* = 153.8 Hz, 1H), 128.40, 128.17, 126.45, 123.36 (d, *J* = 33.7 Hz, 1H), 115.81 (t, *J* = 3.6 Hz), 113.60 (d, *J* = 20.6 Hz, 1H), 111.11 (d, *J* = 5.8 Hz, 1H), 109.60 (t, *J* = 235.3 Hz), 45.73, 39.49. HRMS (ESI): calculated for C_21_H_16_F_3_N_5_O_2_ [M+Na]^+^: 450.11483, found: 450.11509.

3-(difluoromethyl)-*N*-(2-((6-fluoro-4-oxoquinazolin-3(4H)-yl)methyl)phenyl)-1-methyl-1H-pyrazole-4-carboxamide (**6a_8_**), white powder, yield 59%, m.p. 235.4–237.4 °C. ^1^H NMR (400 MHz, DMS*O*-*d_6_*) *δ* 10.19 (s, 1H, *NH*), 8.53 (s, 1H, *pyrazol-H5*), 8.48 (s, 1H, *H2*), 7.84 (dd, *J* = 8.7, 2.8 Hz, 1H, *H5*), 7.81–7.70 (m, 2H, *H7*), 7.46 (dd, *J* = 8.0, 1.3 Hz, 1H, *Ar-H2*), 7.39–7.31 (m, 2H, H8, Ar-*H3*), 7.27 (t, *J* = 54.1 Hz, 1H, CF_2_*H*), 7.26–7.18 (m, 2H, *Ar-H4*, *Ar-H5*), 5.22 (s, 2H, *CH_2_*), 4.01 (s, 3H, *pyrazol-CH_3_*). ^13^C NMR (100 MHz, DMS*O*-*d_6_*) *δ* 160.35 (d, *J* = 245.6 Hz), 160.22, 159.97 (d, *J* = 3.2 Hz), 159.13, 154.86, 147.38, 145.15 (t, *J* = 23.0 Hz), 144.83, 135.36, 132.91, 131.14, 130.8 (d, *J* = 8.4 Hz), 128.41 (d, *J* = 28.2 Hz), 126.39 (d, *J* = 32.0 Hz), 123.10 (d, *J* = 24.1 Hz), 122.71 (d, *J* = 8.7 Hz), 115.83 (t, *J* = 3.7 Hz), 110.86 (d, *J* = 23.5 Hz), 109.60 (t, *J* = 235.3 Hz), 45.98, 39.49. HRMS (ESI): calculated for C_21_H_16_F_3_N_5_O_2_ [M+Na]^+^: 450.11483, found: 450.11509.

3-(difluoromethyl)-*N*-(2-((7-fluoro-4-oxoquinazolin-3(4H)-yl)methyl)phenyl)-1-methyl-1H-pyrazole-4-carboxamide (**6a_9_**), white powder, yield 62%, m.p. 229.8–230.4 °C. ^1^H NMR (400 MHz, DMSO) δ 10.18 (s, 1H, *NH*), 8.53 (s, 1H, *pyrazol-H5*), 8.52 (s, 1H, *H2*), 8.24–8.11 (m, 2H, *H5,H6*), 7.51–7.45 (m, 1H, *H8*), 7.42 (d, *J* = 2.6 Hz, 1H, *Ar-H2*), 7.36–7.32 (m, 1H, Ar-*H3*), 7.29 (t, *J* = 54.1 Hz, 1H, CF_2_*H*), 7.27 (dd, *J* = 7.7, 1.8 Hz, 1H, *Ar-H5*), 7.22 (td, *J* = 7.4, 1.3 Hz, 1H, *Ar-H4*), 5.21 (s, 2H, *CH_2_*), 4.01 (s, 3H, *pyrazol-CH_3_*). ^13^C NMR (100 MHz, DMS*O*-*d_6_*) *δ* 165.71 (d, *J* = 251.6 Hz), 160.22, 159.92, 150.11 (d, *J* = 13.2 Hz), 149.32, 146.94, 145.16 (t, *J* = 23.2 Hz), 135.34, 132.91, 131.22, 129.40 (d, *J* = 10.9 Hz), 128.36(d, *J* = 24.3 Hz), 126.54, 126.23, 118.49, 115.88 (d, *J* = 23.6 Hz), 115.85 (t, *J* = 3.6 Hz), 112.30 (d, *J* = 21.5 Hz),109.61 (t, *J* = 234.7 Hz), 45.93, 39.48. HRMS (ESI): calculated for C_21_H_16_F_3_N_5_O_2_ [M+Na]^+^: 450.11483, found: 450.11509.

3-(difluoromethyl)-*N*-(2-((8-fluoro-4-oxoquinazolin-3(4H)-yl)methyl)phenyl)-1-methyl-1H-pyrazole-4-carboxamide (**6a_10_**), white powder, yield 69%, m.p. 274.8–276.2 °C. ^1^H NMR (400 MHz, DMS*O*-*d_6_*) *δ* 10.13 (s, 1H, *NH*), 8.51 (s, 1H, *pyrazol-H5*), 8.50 (s, 1H, *H2*), 7.95 (dd, *J* = 8.5, 1.3 Hz, 1H, *H5*), 7.76–7.69 (m, 1H, *H7*), 7.57–7.52 (m, 1H, *Ar-H6*), 7.44 (dd, *J* = 7.8, 1.2 Hz, 1H, *Ar-H2*), 7.38–7.31 (m, 1H, Ar-*H3*), 7.27(t, *J* = 54.1 Hz, 1H, CF_2_*H*), 7.27–7.20 (m, 2H, *Ar-H4*, *Ar-H5*), 5.22 (s, 2H, *CH_2_*), 4.00 (s, 3H, *pyrazol-CH_3_*). ^13^C NMR (100 MHz, DMS*O*-*d_6_*) *δ* 160.25, 159.69 (d, *J* = 3.3 Hz), 156.41 (d, *J* = 254.3 Hz), 148.56, 145.15 (t, *J* = 23.2 Hz), 137.16 (d, *J* = 12.3 Hz), 135.29, 132.92, 131.32, 128.34, 128.24, 127.65 (d, *J* = 7.9 Hz), 126.63, 126.32, 123.49, 121.95 (d, *J* = 4.2 Hz), 119.97 (d, *J* = 16.0 Hz), 115.82 (t, *J* = 3.6 Hz), 109.61 (t, *J* = 234.7 Hz), 46.12, 39.72. HRMS (ESI): calculated for C_21_H_16_F_3_N_5_O_2_ [M+Na]^+^: 450.11483, found: 450.11509.

3-(difluoromethyl)-*N*-(2-((6,7-difluoro-4-oxoquinazolin-3(4H)-yl)methyl)phenyl)-1-methyl-1H-pyrazole-4-carboxamide (**6a_11_**), white powder, yield 69%, m.p. 243.1–244.5 °C. ^1^H NMR (400 MHz, DMS*O*-*d_6_*) *δ* 10.15 (s, 1H, *NH*), 8.52 (s, 1H, *pyrazol-H5*), 8.52 (s, 1H, *H2*), 8.06 (dd, *J* = 10.4, 8.6 Hz, 1H, *H5*), 7.78 (dd, *J* = 11.3, 7.3 Hz, 1H, *H8*), 7.46 (dd, *J* = 8.1, 1.3 Hz, 1H, *Ar-H2*), 7.35 (td, *J* = 7.5, 1.7 Hz, 1H, Ar-*H3*), 7.32–7.28 (m, 1H, *Ar-H5*), 7.28(t, *J* = 54.1 Hz, 1H, CF_2_*H*), 7.23 (td, *J* = 7.5, 1.3 Hz, 1H, *Ar-H4*), 5.21 (s, 2H, *CH_2_*), 4.01 (s, 3H, *pyrazol-CH_3_*). ^13^C NMR (100 MHz, DMS*O*-*d_6_*) *δ* 160.22, 159.37 (d, *J* = 2.9 Hz), 153.89 (dd, *J* = 54.6, 14.6 Hz), 148.89 (dd, *J* = 249.1, 14.2 Hz), 148.80 (d, *J* = 2.0 Hz), 145.16 (t, *J* = 23.1 Hz), 135.37, 132.88, 131.05, 128.61, 128.33, 126.59, 126.24, 118.81 (dd, *J* = 6.7, 2.0 Hz), 115.80 (t, *J* = 3.6 Hz), 115.31 (d, *J* = 17.7 Hz), 113.78 (d, *J* = 19.4 Hz), 109.59 (t, *J* = 234.7 Hz), 46.09, 39.72. HRMS (ESI): calculated for C_21_H_16_F_4_N_5_O_2_ [M+Na]^+^: 468.10541, found: 468.10521.

*N*-(2-((5-chloro-4-oxoquinazolin-3(4H)-yl)methyl)phenyl)-3-(difluoromethyl)-1-methyl-1H-pyrazole-4-carboxamide (**6a_12_**), white powder, yield 58%, m.p. 172.8–174.7 °C. ^1^H NMR (400 MHz, DMS*O*-*d_6_*) *δ* 10.14 (s, 1H, *NH*), 8.53 (s, 1H, *pyrazol-H5*), 8.49 (s, 1H, *H2*), 7.78–7.70 (m, 1H, *H7*), 7.62 (dt, *J* = 8.2, 1.4 Hz, 1H, *H6*), 7.56 (dq, *J* = 7.8, 1.2 Hz, 1H, *H8*), 7.46 (dt, *J* = 8.0, 1.4 Hz, 1H, *Ar-H2*), 7.42–7.29 (m, 2H, CF_2_*H, Ar-H3*), 7.28–7.11 (m, 2H, *Ar-H4*, *Ar-H5*), 5.16 (s, 2H, *CH_2_*), 3.99 (s, 3H, *pyrazol-CH_3_*). ^13^C NMR (100 MHz, DMS*O*-*d_6_*) *δ* 160.22, 158.57, 150.42, 148.65, 145.14 (t, *J* = 23.0 Hz), 135.44, 134.37, 132.98, 132.50, 131.25, 129.71, 128.69, 128.27, 126.92, 126.44, 126.21, 118.50, 115.83 (t, *J* = 3.7 Hz), 109.59 (t, *J* = 234.7 Hz), 46.08, 39.70. HRMS (ESI): calculated for C_21_H_16_ClF_2_N_5_O_2_ [M+Na]^+^: 446.08528, found: 466.08523.

*N*-(2-((6-chloro-4-oxoquinazolin-3(4H)-yl)methyl)phenyl)-3-(difluoromethyl)-1-methyl-1H-pyrazole-4-carboxamide (**6a_13_**), white powder, yield 66%, m.p. 259.1–260.4 °C. ^1^H NMR (400 MHz, DMS*O*-*d_6_*) *δ* 10.36 (s, 1H, *NH*), 8.69 (s, 1H, *pyrazol-H5*), 8.52 (s, 1H, *H2*), 8.07 (d, *J* = 2.5 Hz, 1H, *H5*), 7.85 (dd, *J* = 8.8, 2.5 Hz, 1H, *H7*), 7.71 (d, *J* = 8.7 Hz, 1H, *H8*), 7.43 (dd, *J* = 8.0, 1.3 Hz, 1H, *Ar-H2*), 7.36–7.31 (m, 1H, Ar-*H3*), 7.26(t, *J* = 54.1 Hz, 1H, CF_2_*H*), 7.25–7.18 (m, 2H, *Ar-H4*, *Ar-H5*), 5.26 (s, 2H, *CH_2_*), 4.00 (s, 3H, *pyrazol-CH_3_*). ^13^C NMR (100 MHz, DMS*O*-*d_6_*) *δ* 160.29, 159.56, 148.40, 146.63, 145.15 (t, *J* = 23.0 Hz), 135.39, 134.63, 133.15, 131.52, 131.31, 130.98, 128.51, 128.16, 126.86, 126.20, 125.13, 122.72, 115.81 (t, *J* = 3.7 Hz), 109.59 (t, *J* = 234.7 Hz), 46.14, 39.52. HRMS (ESI): calculated for C_21_H_16_ClF_2_N_5_O_2_ [M+Na]^+^: 446.08528, found: 466.08523.

*N*-(2-((7-chloro-4-oxoquinazolin-3(4H)-yl)methyl)phenyl)-3-(difluoromethyl)-1-methyl-1H-pyrazole-4-carboxamide (**6a_14_**), white powder, yield 63%, m.p. 250.3–252.3 °C. ^1^H NMR (400 MHz, DMS*O*-*d_6_*) *δ* 10.14 (s, 1H, *NH*), 8.51 (s, 1H, *pyrazol-H5*), 8.50 (s, 1H, *H2*), 8.14 (d, *J* = 8.6 Hz, 1H, *H5*), 7.76 (d, *J* = 2.1 Hz, 1H, *H8*), 7.59 (dd, *J* = 8.5, 2.1 Hz, 1H, *H6*), 7.45 (dd, *J* = 7.9, 1.3 Hz, 1H, *Ar-H2*), 7.35 (td, *J* = 7.5, 1.9 Hz, 1H, Ar-*H3*),7.28 (t, *J* = 54.1 Hz, 1H, CF_2_*H*), 7.27–7.19 (m, 2H, *Ar-H4*, *Ar-H5*), 5.20 (s, 2H, *CH_2_*), 4.00 (s, 3H, *pyrazol-CH_3_*). ^13^C NMR (100 MHz, DMS*O*-*d_6_*) *δ* 160.22, 160.00, 149.38, 149.01, 145.13 (t, *J* = 23.2 Hz), 139.21, 135.31, 132.88, 131.24, 128.45, 128.29, 128.26, 127.55, 126.59, 126.42, 126.27, 120.29, 115.82(t, *J* = 3.6 Hz), 109.60 (t, *J* = 234.6 Hz), 46.02, 39.75. HRMS (ESI): calculated for C_21_H_16_ClF_2_N_5_O_2_ [M+Na]^+^: 446.08528, found: 466.08523.

*N*-(2-((8-chloro-4-oxoquinazolin-3(4H)-yl)methyl)phenyl)-3-(difluoromethyl)-1-methyl-1H-pyrazole-4-carboxamide (**6a_15_**), white powder, yield 68%, m.p. 231.8–233.0 °C. ^1^H NMR (400 MHz, DMS*O*-*d_6_*) *δ* 10.13 (s, 1H, *NH*), 8.58 (s, 1H, *pyrazol-H5*), 8.50 (s, 1H, *H2*), 8.10 (dd, *J* = 7.7, 1.2 Hz, 1H, *H5*), 7.99 (dd, *J* = 7.8, 1.4 Hz, 1H, *H7*), 7.56–7.50 (m, 1H, *H6*), 7.44 (dd, *J* = 7.9, 1.3 Hz, 1H, *Ar-H2*), 7.35 (ddd, *J* = 8.1, 6.7, 2.2 Hz, 1H, Ar-*H3*), 7.29(t, *J* = 54.1 Hz, 1H, CF_2_*H*), 7.28–7.20 (m, 2H, *Ar-H4*, *Ar-H5*), 5.22 (s, 2H, *CH_2_*), 4.00 (s, 3H, *pyrazol-CH_3_*). ^13^C NMR (100 MHz, DMS*O*-*d_6_*) *δ* 160.25, 160.01, 148.86, 145.24, 145.15 (t, *J* = 23.2 Hz), 144.40, 135.27, 134.62, 132.93, 131.28, 128.31, 128.23, 127.65, 126.62, 126.33, 125.37, 123.20, 115.80 (t, *J* = 3.7 Hz), 109.61 (t, *J* = 235.4 Hz), 46.17, 39.72. HRMS (ESI): calculated for C_21_H_16_ClF_2_N_5_O_2_ [M+Na]^+^: 446.08528, found: 466.08523.

*N*-(2-((6,8-dichloro-4-oxoquinazolin-3(4H)-yl)methyl)phenyl)-3-(difluoromethyl)-1-methyl-1H-pyrazole-4-carboxamide (**6a_16_**), white powder, yield 77%, m.p. 294.1–294.7 °C. ^1^H NMR (400 MHz, DMS*O*-*d_6_*) *δ* 10.22 (s, 1H, *NH*), 8.60 (s, 1H, *pyrazol-H5*), 8.59 (s, 1H, *H2*), 8.12 (d, *J* = 2.4 Hz, 1H, *H5*), 7.41 (dd, *J* = 8.1, 1.5 Hz, 1H, *H7*), 7.34 (td, *J* = 7.4, 2.0 Hz, 1H, *Ar-H2*), 7.27–7.20 (m, 3H, CF_2_*H*, *Ar-H4*, *Ar-H5*) 5.23 (s, 2H, *CH_2_*), 4.00 (s, 3H, *pyrazol-CH_3_*). ^13^C NMR (100 MHz, DMS*O*-*d_6_*) *δ* 160.29, 159.61, 150.97, 149.19, 147.16, 146.02 (t, *J* = 23.2 Hz), 144.33, 133.86, 132.30, 131.21, 130.53, 128.41, 128.23, 127.44, 125.02, 124.43, 124.15, 116.16 (t, *J* = 3.7 Hz), 108.33 (t, *J* = 235.7 Hz), 55.69, 39.52. HRMS (ESI): calculated for C_21_H_15_Cl_2_F_2_N_5_O_2_ [M+Na]^+^: 500.04631, found: 500.04620.

*N*-(2-((6-chloro-8-methyl-4-oxoquinazolin-3(4H)-yl)methyl)phenyl)-3-(difluoromethyl)-1-methyl-1H-pyrazole-4-carboxamide (**6a_17_**), white powder, yield 69%, m.p. >310 °C. ^1^H NMR (400 MHz, DMS*O*-*d_6_*) *δ* 10.29 (s, 1H, *NH*), 8.62 (s, 1H, *pyrazol-H5*), 8.55 (s, 1H, *H2*), 7.92 (dd, *J* = 2.6, 0.7 Hz, 1H, *H5*), 7.76 (dd, *J* = 2.5, 1.0 Hz, 1H, *H7*), 7.44 (dd, *J* = 8.0, 1.2 Hz, 1H, *Ar-H2*), 7.36–7.31 (m, 1H, Ar-*H3*),7.27 (t, *J* = 54.1 Hz, 1H, CF_2_*H*), 7.26–7.18 (m, 2H, *Ar-H4*, *Ar-H5*), 5.23 (s, 2H, *CH_2_*), 3.99 (s, 3H, *pyrazol-CH_3_*), 2.51 (s, 3H, *8-CH_3_*). ^13^C NMR (100 MHz, DMS*O*-*d_6_*) *δ* 160.25, 159.82, 147.45, 145.27, 145.14 (t, *J* = 23.2 Hz), 138.57, 135.33, 134.54, 133.07, 131.24, 130.98, 128.43, 128.17, 126.73, 126.22, 122.65, 122.57, 115.81 (t, *J* = 3.4 Hz), 109.60 (t, *J* = 235.3 Hz), 46.10, 39.73. 16.77. HRMS (ESI): calculated for C_22_H_18_ClF_2_N_5_O_2_ [M+Na]^+^: 480.10093, found:480.10045.

*N*-(2-((5-bromo-4-oxoquinazolin-3(4H)-yl)methyl)phenyl)-3-(difluoromethyl)-1-methyl-1H-pyrazole-4-carboxamide (**6a_18_**), gray powder, yield 69%, m.p. 274.8–276.2 °C. ^1^H NMR (400 MHz, DMS*O*-*d_6_*) *δ* 10.17 (s, 1H, *NH*), 8.55 (s, 1H, *pyrazol-H5*), 8.52 (s, 1H, *H2*), 7.78 (dd, *J* = 6.6, 2.3 Hz, 1H, *H6*), 7.70–7.62 (m, 2H, *H7*, *H8*), 7.47 (dd, *J* = 7.9, 1.4 Hz, 1H, *Ar-H2*), 7.38–7.31 (m, 2H, *Ar-H3, Ar-H5*),7.27 (t, *J* = 54.1 Hz, 1H, CF_2_*H*), 7.24 (td, *J* = 7.5, 1.4 Hz, 1H, *Ar-H4*), 5.16 (s, 2H, *CH_2_*), 3.99 (s, 3H, *pyrazol-CH_3_*). ^13^C NMR (100 MHz, DMS*O*-*d_6_*) *δ* 160.18, 158.72, 150.26, 148.43, 145.13 (t, *J* = 23.2 Hz), 135.46, 134.68, 133.52, 132.94, 131.07, 128.78, 128.31, 127.61, 126.33, 126.16, 120.15, 119.37, 115.84 (t, *J* = 3.6 Hz), 109.59 (t, *J* = 234.7 Hz), 46.21, 39.54. HRMS (ESI): calculated for C_21_H_16_BrF_2_N_5_O_2_ [M+Na]^+^: 510.03476, found: 510.03443.

*N*-(2-((6-bromo-4-oxoquinazolin-3(4H)-yl)methyl)phenyl)-3-(difluoromethyl)-1-methyl-1H-pyrazole-4-carboxamide (**6a_19_**), white powder, yield 41%, m.p. 281.6–282.3 °C. ^1^H NMR (400 MHz, DMS*O*-*d_6_*) *δ* 10.13 (s, 1H, *NH*), 8.51 (s, 1H, *pyrazol-H5*), 8.50 (s, 1H, *H2*), 8.23 (d, *J* = 2.4 Hz, 1H, *H5*), 7.99 (dd, *J* = 8.7, 2.4 Hz, 1H, *H7*), 7.65 (d, *J* = 8.7 Hz, 1H, *H8*), 7.44 (dd, *J* = 8.0, 1.3 Hz, 1H, *Ar-H2*), 7.35 (td, *J* = 7.5, 2.0 Hz, 1H, Ar-*H3*), 7.27 (t, *J* = 54.1 Hz, 1H, CF_2_*H*), 7.26–7.20 (m, 2H, *Ar-H4*, *Ar-H5*), 5.21 (s, 2H, *CH_2_*), 4.01 (s, 3H, *pyrazol-CH_3_*). ^13^C NMR (100 MHz, DMS*O-d6*) *δ* 160.22, 159.46, 148.50, 146.92, 145.13 (t, *J* = 23.0 Hz), 144.90, 137.42, 135.30, 132.88, 131.23, 129.67, 128.45, 128.26, 126.65, 126.29, 123.05, 119.77, 115.79(t, *J* = 3.4 Hz), 109.59(t, *J* = 235.3 Hz), 46.08, 39.73. HRMS (ESI): calculated for C_21_H_16_BrF_2_N_5_O_2_ [M+Na]^+^: 510.03476, found: 510.03443.

*N*-(2-((7-bromo-4-oxoquinazolin-3(4H)-yl)methyl)phenyl)-3-(difluoromethyl)-1-methyl-1H-pyrazole-4-carboxamide (**6a_20_**), grey powder, yield 67%, m.p. 262.8–263.9 °C. ^1^H NMR (400 MHz, DMS*O*-*d_6_*) *δ* 10.14 (s, 1H, *NH*), 8.50 (s, 1H, *pyrazol-H5*), 8.49 (s, 1H, *H2*), 8.05 (dd, *J* = 8.5, 1.1 Hz, 1H, *H5*), 7.95–7.87 (m, 1H, *H6*), 7.75–7.67 (m, 1H, *H8*), 7.45 (dd, *J* = 8.0, 1.9 Hz, 1H, *Ar-H2*), 7.38–7.31 (m, 1H Ar-*H3*), 7.30–7.12 (m, 3H, CF_2_*H, Ar-H4, Ar-H5*), 5.20 (s, 2H, *CH_2_*), 4.00 (s, 3H, *pyrazol-CH_3_*). ^13^C NMR (100 MHz, DMS*O*-*d_6_*) *δ* 160.22, 160.14, 149.32, 149.05, 145.14 (t, *J* = 23.2 Hz), 135.31, 132.89, 131.22, 130.30, 129.51, 128.46, 128.26, 128.26, 128.18, 126.59, 126.28, 120.58, 115.85 (d, *J* = 3.7 Hz), 109.60 (t, *J* = 234.6 Hz), 46.04, 39.73. HRMS (ESI): calculated for C_21_H_16_BrF_2_N_5_O_2_ [M+Na]^+^: 510.03476, found: 510.03443.

*N*-(2-((8-bromo-4-oxoquinazolin-3(4H)-yl)methyl)phenyl)-3-(difluoromethyl)-1-methyl-1H-pyrazole-4-carboxamide (**6a_21_**), white powder, yield 59%, m.p. 263.7–266.1 °C. ^1^H NMR (400 MHz, DMS*O*-*d_6_*) *δ* 10.13 (s, 1H, *NH*), 8.58 (s, 1H, *pyrazol-H5*), 8.50 (s, 1H, *H2*), 8.18–8.13 (m, 2H, *H5, H7*), 7.49–7.43 (m, 2H, *H6, Ar-H2*), 7.39–7.31 (m, 1H, Ar-*H3*), 7.27 (t, *J* = 54.1 Hz, 1H, CF_2_*H*), 7.28–7.20 (m, 2H*, Ar-H4, Ar-H5*), 5.21 (s, 2H, *CH_2_*), 4.00 (s, 3H, *pyrazol-CH_3_*). ^13^C NMR (100 MHz, DMS*O*-*d_6_*) *δ* 160.26, 160.00, 148.93, 145.42 (t, *J* = 23.2 Hz), 145.14, 137.95, 135.27, 132.93, 131.28, 128.30, 128.23, 128.12, 126.61, 126.33, 126.07, 123.10, 121.81, 115.80(t, *J* = 3.6 Hz), 109.61 (t, *J* = 234.7 Hz), 46.18, 39.73. HRMS (ESI): calculated for C_21_H_16_BrF_2_N_5_O_2_ [M+Na]^+^: 510.03476, found: 510.03443.

*N*-(2-((6,8-dibromo-4-oxoquinazolin-3(4H)-yl)methyl)phenyl)-3-(difluoromethyl)-1-methyl-1H-pyrazole-4-carboxamide (**6a_22_**), white powder, yield 57%, m.p. >310 °C. ^1^H NMR (400 MHz, DMS*O*-*d_6_*) *δ* 10.08 (s, 1H, *NH*), 8.60 (s, 1H, *pyrazol-H5*), 8.48 (s, 1H, *H2*), 8.38 (d, *J* = 2.2 Hz, 1H, *H5*), 8.20 (d, *J* = 2.2 Hz, 1H, *H7*), 7.41 (dd, *J* = 8.0, 1.3 Hz, 1H, *Ar-H2*), 7.35 (ddd, *J* = 8.0, 6.7, 2.0 Hz, 1H, Ar-*H3*), 7.27 (t, *J* = 54.1 Hz, 1H, CF_2_*H*), 7.26–7.20 (m, 2H, *Ar-H4*, *Ar-H5*), 5.20 (s, 2H, *CH_2_*), 4.00 (s, 3H, *pyrazol-CH_3_*). ^13^C NMR (100 MHz, DMS*O*-*d_6_*) *δ* 160.24, 158.95, 149.35, 145.12 (t, *J* = 23.2 Hz), 144.70, 139.68, 135.24, 132.91, 131.22, 128.36, 128.28, 128.15, 126.80, 126.39, 124.05, 123.33, 119.55, 115.72 (t, *J* = 3.7 Hz), 109.57 (t, *J* = 235.7 Hz), 46.39, 39.72. HRMS (ESI): calculated for C_21_H_16_Br_2_F_2_N_5_O_2_ [M+Na]^+^: 587.94528, found: 587.94462.

3-(difluoromethyl)-*N*-(2-((5-methoxy-4-oxoquinazolin-3(4H)-yl)methyl)phenyl)-1-methyl-1H-pyrazole-4-carboxamide (**6a_23_**), white powder, yield 68%, m.p. 212.9–214.8 °C. ^1^H NMR (400 MHz, DMS*O*-*d_6_*) *δ* 10.24 (s, 1H, *NH*), 8.57 (s, 1H, *pyrazol-H5*), 8.42 (s, 1H, *H2*), 7.72 (t, *J* = 8.2 Hz, 1H, *H7*), 7.50 (dd, *J* = 8.0, 1.4 Hz, 1H, *Ar-H2*), 7.34 (td, *J* = 7.6, 1.7 Hz, 1H, Ar-*H3*), 7.30 (t, *J* = 54.1 Hz, 1H, CF_2_*H*), 7.29–7.26 (m, 1H, *Ar-H5*), 7.24–7.17 (m, 2H, *H8, Ar-H4*), 7.05 (dd, *J* = 8.4, 1.0 Hz, 1H, *H6*), 5.12 (s, 2H, *CH_2_*), 4.01 (s, 3H, *pyrazol-CH_3_*), 3.85 (s, 3H, *5-CH_3_O*). ^13^C NMR (100 MHz, DMS*O*-*d_6_*) *δ* 160.16, 159.71, 158.51, 150.49, 148.22, 145.37 (t, *J* = 23.2 Hz), 135.44, 135.01, 132.96, 131.14, 128.66, 128.15, 125.99, 124.39, 119.00, 115.92 (t, *J* = 3.7 Hz), 109.78 (t, *J* = 235.4 Hz), 56.13, 45.57, 39.72. HRMS (ESI): calculated for C_22_H_19_F_2_N_5_O_3_ [M+Na]^+^: 439.14560, found: 462.13426.

3-(difluoromethyl)-*N*-(2-((6-methoxy-4-oxoquinazolin-3(4H)-yl)methyl)phenyl)-1-methyl-1H-pyrazole-4-carboxamide (**6a_24_**), white powder, yield 70%, m.p. 259.8–260.2 °C. ^1^H NMR (400 MHz, DMS*O*-*d_6_*) *δ* 10.30 (s, 1H, *NH*), 8.64 (s, 1H, *pyrazol-H5*), 8.38 (s, 1H, *H2*), 7.64 (d, *J* = 8.9 Hz, 1H, *H5*), 7.52 (d, *J* = 3.0 Hz, 1H, *H8*), 7.50–7.43 (m, 2H, *H6 Ar-H2*), 7.38–7.31 (m, 1H, Ar-*H3*), 7.30 (t, *J* = 54.1 Hz, 1H, CF_2_*H*), 7.27–7.17 (m, 2H, *Ar-H4*, *Ar-H5*), 5.25 (s, 2H, *CH_2_*), 4.00 (s, 3H, *pyrazol-CH_3_*), 3.86 (s, 3H, *6-CH_3_O*). ^13^C NMR (100 MHz, DMS*O*-*d_6_*) *δ* 160.32, 160.22, 158.12, 145.67, 145.15 (t, *J* = 23.2 Hz), 135.35, 133.07, 131.34, 128.96, 128.40, 128.11, 126.56, 126.13, 124.04, 122.28, 115.89 (t, *J* = 3.7 Hz), 109.63 (t, *J* = 235.4 Hz), 106.10, 55.66, 45.84, 39.52. HRMS (ESI): calculated for C_22_H_19_F_2_N_5_O_3_ [M+Na]^+^: 439.14560, found: 462.13426.

3-(difluoromethyl)-*N*-(2-((7-methoxy-4-oxoquinazolin-3(4H)-yl)methyl)phenyl)-1-methyl-1H-pyrazole-4-carboxamide (**6a_25_**), white powder, yield 69%, m.p. 254.1–276.3 °C. ^1^H NMR (400 MHz, DMS*O*-*d_6_*) *δ* 10.43 (s, 1H, *NH*), 8.70 (s, 1H, *pyrazol-H5*), 8.50 (s, 1H, *H2*), 8.07 (d, *J* = 9.6 Hz, 1H, *H5*), 7.49 (dd, *J* = 8.0, 1.3 Hz, 1H, *Ar-H2*), 7.33 (td, *J* = 7.5, 1.8 Hz, 1H, Ar-*H3*), 7.30 (t, *J* = 54.1 Hz, 1H, CF_2_*H*), 7.26 (dd, *J* = 7.8, 1.7 Hz, 1H, Ar-*H5*), 7.20 (td, *J* = 7.5, 1.3 Hz, 1H, Ar-*H4*), 7.15–7.11 (m, 2H, *H6, H8*), 5.23 (s, 2H, *CH_2_*), 4.00 (s, 3H, *pyrazol-CH_3_*), 3.89 (s, 3H, *7-CH_3_O*). ^13^C NMR (100 MHz, DMS*O*-*d_6_*) *δ* 164.15, 160.20, 160.18, 150.26, 148.52, 145.16 (t, *J* = 23.2 Hz), 135.41, 133.09, 131.18, 128.55, 128.11, 127.85, 126.42, 126.01, 116.86, 115.93 (t, *J* = 3.7 Hz), 114.80, 109.64 (t, *J* = 235.4 Hz), 108.29, 55.82, 45.66, 39.52. HRMS (ESI): calculated for C_22_H_19_F_2_N_5_O_3_ [M+Na]^+^: 439.14560, found: 462.13426.

3-(difluoromethyl)-*N*-(2-((8-methoxy-4-oxoquinazolin-3(4H)-yl)methyl)phenyl)-1-methyl-1H-pyrazole-4-carboxamide (**6a_26_**), white powder, yield 70%, m.p. 268.8–270.4 °C. ^1^H NMR (400 MHz, DMS*O*-*d_6_*) *δ* 10.22 (s, 1H, *NH*), 8.54 (s, 1H, *pyrazol-H5*), 8.44 (s, 1H, *H2*), 7.71 (dd, *J* = 8.0, 1.3 Hz, 1H, *H5*), 7.52–7.43 (m, 2H, *H6*, Ar-*H2*), 7.41–7.32 (m, 2H, *H7,* Ar-*H3*), 7.31 (t, *J* = 54.1 Hz, 1H, CF_2_*H*), 7.29–7.17 (m, 2H, Ar-*H4,* Ar-*H5*), 5.22 (s, 2H, *CH_2_*), 4.01 (s, 3H, *pyrazol-CH_3_*), 3.90 (s, 3H, *8-CH_3_O*). ^13^C NMR (100 MHz, DMS*O*-*d_6_*) *δ* 160.51, 160.17, 154.53, 146.49, 145.16 (t, *J* = 23.2 Hz), 138.45, 135.33, 132.90, 131.14, 128.35, 128.18, 127.68, 126.34, 126.14, 122.46, 117.07, 115.89 (t, *J* = 3.7 Hz), 115.08, 109.65 (t, *J* = 235.4 Hz), 56.01, 45.82, 39.52. HRMS (ESI): calculated for C_22_H_19_F_2_N_5_O_3_ [M+Na]^+^: 439.14560, found: 462.13426.HRMS (ESI): calculated for C_22_H_19_F_2_N_5_O_3_ [M+Na]^+^: 439.14560, found: 462.13426.

3-(difluoromethyl)-*N*-(2-((6,7-dimethoxy-4-oxoquinazolin-3(4H)-yl)methyl)phenyl)-1-methyl-1H-pyrazole-4-carboxamide (**6a_27_**), white powder, yield 77%, m.p. 290.3–292.5 °C. ^1^H NMR (400 MHz, DMS*O*-*d_6_*) *δ* 10.26 (s, 1H, *NH*), 8.55 (s, 1H, *pyrazol-H5*), 8.41 (s, 1H, *H2*), 7.51 (dd, *J* = 8.0, 1.3 Hz, 1H, Ar-*H2*), 7.46 (s, 1H, *H5*), 7.34 (td, *J* = 7.6, 1.8 Hz, 1H, Ar-*H3*), 7.31 (t, *J* = 54.1 Hz, 1H, CF_2_*H*), 7.26 (dd, *J* = 7.8, 1.8 Hz, 1H, Ar-*H5*), 7.21 (ddd, *J* = 7.8, 7.1, 1.3 Hz, 1H, Ar-*H4*), 7.16 (s, 1H, *H8*), 5.21 (s, 2H, *CH_2_*), 4.01 (s, 3H, *pyrazol-CH_3_*), 3.91 (s, 3H, *7-CH_3_O*), 3.87 (s, 3H, *6-CH_3_O*). ^13^C NMR (100 MHz, DMS*O*-*d_6_*) *δ* 160.13, 159.94, 154.80, 149.00, 146.37, 145.15 (t, *J* = 23.2 Hz), 144.22, 135.36, 132.90, 131.05, 128.54, 128.20, 126.17, 126.03, 115.95 (t, *J* = 3.7 Hz), 114.40, 109.64 (t, *J* = 235.7 Hz), 107.92, 105.12, 56.05, 55.76, 45.68, 39.72. HRMS (ESI): calculated for C_23_H_21_F_2_N_5_O_4_ [M+Na]^+^: 492.14538, found: 492.14539.

3-(difluoromethyl)-1-methyl-*N*-(2-((5-nitro-4-oxoquinazolin-3(4H)-yl)methyl)phenyl)-1H-pyrazole-4-carboxamide (**6a_28_**), white powder, yield 75%, m.p. 278.1–279.2 °C. ^1^H NMR (400 MHz, DMS*O*-*d_6_*) *δ* 10.09 (s, 1H, *NH*), 8.61 (s, 1H, *H2*), 8.43 (s, 1H, *pyrazol-H5*), 8.02–7.98 (m, 1H, *H6*), 7.94–7.91 (m, 1H, *H8*), 7.83–7.79 (m, 1H, *H7*), 7.46 (dd, *J* = 7.9, 1.3 Hz, 1H, Ar-*H2*), 7.39–7.32 (m, 2H, Ar-*H3*, Ar-*H5*), 7.27 (t, *J* = 54.1 Hz, 1H, CF_2_*H*), 7.24 (td, *J* = 7.5, 1.4 Hz, 1H, Ar-*H4*), 5.18 (s, 2H, *CH_2_*), 4.01 (s, 3H, *pyrazol-CH_3_*). ^13^C NMR (100 MHz, DMS*O*-*d_6_*) *δ* 160.24, 157.45, 157.18, 149.69, 148.15, 145.15(t, *J* = 23.2 Hz), 135.50, 134.99, 132.91, 130.75, 130.15, 130.04, 128.92, 128.48, 126.42, 126.24, 120.99, 115.76(t, *J* = 3.6 Hz), 109.58 (t, *J* = 234.7 Hz), 46.17, 39.47. HRMS (ESI): calculated for C_21_H_16_F_2_N_6_O_4_ [M+Na]^+^: 477.10933, found: 477.10936.

3-(difluoromethyl)-1-methyl-*N*-(2-((6-nitro-4-oxoquinazolin-3(4H)-yl)methyl)phenyl)-1H-pyrazole-4-carboxamide (**6a_29_**), light yellow powder, yield 64%, m.p. 233.9–234.7 °C. ^1^H NMR (400 MHz, DMS*O*-*d_6_*) *δ* 10.07 (s, 1H, *NH*), 8.77 (d, *J* = 2.7 Hz, 1H, *H5*), 8.63 (s, 1H, *pyrazol-H5*), 8.55 (dd, *J* = 9.0, 2.7 Hz, 1H, *H7*), 8.49 (s, 1H, *H2*), 7.88 (d, *J* = 9.0 Hz, 1H, *H8*), 7.40 (dd, *J* = 7.9, 1.6 Hz, 1H, Ar-*H2*), 7.36 (td, *J* = 7.2, 1.8 Hz, 1H, Ar-*H3*), 7.31–7.27 (m, 1H, Ar-*H5*), 7.27 (t, *J* = 54.1 Hz, 1H, CF_2_*H*), 7.27–7.22 (m, 1H, Ar-*H4*), 5.25 (s, 2H, *CH_2_*), 4.00 (s, 3H, *pyrazol-CH_3_*). ^13^C NMR (100 MHz, DMS*O*-*d_6_*) *δ* 160.29, 159.73, 151.97, 151.22, 145.31, 145.11 (t, *J* = 23.2 Hz), 135.28, 132.92, 131.45, 129.15, 128.45, 128.39, 128.33, 127.07, 126.48, 122.22, 121.64, 115.68 (t, *J* = 3.7 Hz), 109.53 (t, *J* = 235.7 Hz), 46.35, 39.52. HRMS (ESI): calculated for C_21_H_16_F_2_N_6_O_4_ [M+Na]^+^: 477.10933, found: 477.10936.

3-(difluoromethyl)-1-methyl-*N*-(2-((7-nitro-4-oxoquinazolin-3(4H)-yl)methyl)phenyl)-1H-pyrazole-4-carboxamide (**6a_30_**), orange powder, yield 61%, m.p. 253.8–256.1 °C. ^1^H NMR (400 MHz, DMS*O*-*d_6_*) *δ* 10.09 (s, 1H, *NH*), 8.60 (s, 1H, *pyrazol-H5*), 8.48 (s, 1H, *H2*), 8.41 (d, *J* = 2.2 Hz, 1H, *H8*), 8.34 (dd, *J* = 8.8, 0.5 Hz, 1H, *H5*), 8.26 (dd, *J* = 8.8, 2.3 Hz, 1H, *H6*), 7.41 (dd, *J* = 7.9, 1.4 Hz, 1H, Ar-*H2*), 7.36 (td, *J* = 7.4, 1.9 Hz, 1H, Ar-*H3*), 7.28 (dd, *J* = 7.8, 1.9 Hz, 1H, Ar-*H5*), 7.26–7.21 (m, 2H, CF_2_*H*, Ar-*H4*), 5.23 (s, 2H, *CH_2_*), 4.00 (s, 3H, *pyrazol-CH_3_*). ^13^C NMR (100 MHz, DMS*O*-*d_6_*) *δ* 160.29, 159.59, 151.14, 150.16, 148.27, 145.12 (t, *J* = 23.2 Hz), 135.30, 132.91, 131.35, 128.53, 128.43, 128.31, 126.88, 126.42, 125.81, 122.33, 120.83, 115.78 (t, *J* = 3.7 Hz), 109.57 (t, *J* = 235.7 Hz), 46.35, 39.72. HRMS (ESI): calculated for C_21_H_16_F_2_N_6_O_4_ [M+Na]^+^: 477.10933, found: 477.10936.

3-(difluoromethyl)-1-methyl-*N*-(2-((8-nitro-4-oxoquinazolin-3(4H)-yl)methyl)phenyl)-1H-pyrazole-4-carboxamide (**6a_31_**), yellow powder, yield 60%, m.p. 226.8–208.0 °C. ^1^H NMR (400 MHz, DMS*O-d6*) *δ* 10.09 (s, 1H, *NH*), 8.59 (s, 1H, *pyrazol-H5*), 8.48 (s, 1H, *H2*), 8.36 (d, *J* = 8.0 Hz, 1H, *H5*), 8.33 (d, *J* = 7.9 Hz, 1H, *H7*), 7.69 (t, *J* = 7.9 Hz, 1H, *H6*), 7.46–7.38 (m, 1H, Ar-*H2*), 7.37–7.32 (m, 1H, Ar-*H3*), 7.31–7.20 (m, 3H, CF_2_*H*, Ar-*H4*, Ar-*H5*), 5.22 (s, 2H, *CH_2_*), 4.00 (s, 3H, *pyrazol-CH_3_*). ^13^C NMR (100 MHz, DMSO*-d_6_*) *δ* 160.29, 159.13, 150.56, 146.48, 145.14 (t, *J* = 23.2 Hz), 139.66, 135.26, 132.95, 131.35, 129.96, 128.39, 128.28, 127.98, 126.94, 126.83, 126.44, 122.96, 115.74 (t, *J* = 3.7 Hz), 109.59 (t, *J* = 235.7 Hz), 46.43, 39.52. HRMS (ESI): calculated for C_21_H_16_F_2_N_6_O_4_ [M+Na]^+^: 477.10933, found: 477.10936.

3-(difluoromethyl)-*N*-(2-((6-iodo-4-oxoquinazolin-3(4H)-yl)methyl)phenyl)-1-methyl-1H-pyrazole-4-carboxamide (6a_32_), white powder, yield 74%, m.p. 261.8–263.1 °C. ^1^H NMR (400 MHz, DMS*O*-*d_6_*) *δ* 10.12 (s, 1H, *NH*), 8.49 (s, 1H, *pyrazol-H5*), 8.49 (s, 1H, *H2*), 8.41 (d, *J* = 2.1 Hz, 1H, *H5*), 8.14–8.07 (m, 1H, *H7*), 7.50–7.44 (m, 2H, *H8, Ar-H2*), 7.39–7.30 (m, 1H, Ar-*H3*), 7.27 (t, *J* = 54.1 Hz, 1H, CF_2_*H*), 7.26–7.19 (m, 2H, Ar-*H5*, Ar-*H5*), 5.20 (s, 2H, *CH_2_*), 4.01 (s, 3H, *pyrazol-CH_3_*). ^13^C NMR (100 MHz, DMS*O*-*d_6_*) *δ* 160.21, 159.26, 148.53, 147.19, 145.13(t, *J* = 23.2 Hz), 142.92, 135.27, 134.40, 132.88, 131.27, 129.40, 128.39, 128.24, 126.65, 126.29, 123.20, 115.80 (t, *J* = 3.7 Hz), 109.59 (t, *J* = 235.7 Hz), 92.48, 46.06, 39.73. HRMS (ESI): calculated for C_21_H_16_F_2_IN_5_O_2_ [M+Na]^+^: 558.02090, found: 558.02095.

### 2.2. X-ray Diffraction

Compound **6a_11_** was recrystallized through slow evaporation from a tetrahydrofuran/*N*-hexane (υ/υ = 2:1) solution to produce a single crystal that was suitable for X-ray crystallography for structure validation.

### 2.3. In Vitro Antifungal Assay

*R. solani* was the test strain. It was provided by the Guizhou Institute of Plant Protection. In this study, the in vitro antifungal activity of the target compound **6a_1_-a_32_** against *R. solani* was screened using the mycelial growth rate method [27,28]. The commercial fungicides fluconazole and bixafen were selected as the positive controls. *R. solani* was inoculated on potato dextrose agar (PDA) plates and grown in biochemical incubators at 28 ± 1 °C for 2 days. The newly grown mycelia were used to determine the antifungal activity. The tested compounds were dissolved in DMSO to prepare 10 mg/mL stock solutions before mixing with PDA. The PDA containing the test compounds at a concentration of 100 mg/L was then poured into sterile Petri dishes for primary screening. A data processing system (DPS, V9.50) was used for statistical analysis of the test data, and the significant differences were determined using Duncan’s new multiple range method. The EC_50_ values and 95% confidence limits were calculated after testing the inhibition rates, based on Duncan’s new multiple range method. The inhibition rate of the potent compounds was further tested and the corresponding EC_50_ values were calculated using DPS. The relative inhibitory rates of the potent compounds were then calculated using the following equation:

Inhibition rate (%) = [(C − T)/(C − 5)] × 100%

where C is the colony diameter of the control (mm), T is the colony diameter of the treatment (mm), and 5 is the diameter of the mycelium disks.

### 2.4. SEM Observations

SEM observations of *R. solani* hyphae were conducted following the reported methods [29,30]. Mycelium disks with a diameter of 5 mm were taken from the edge of the PDA medium containing 12.29 mg/L of **6a_16_** and were incubated for 2 days at 28 ± 1 °C. Mycelium disks from PDA with 0.1% DMSO were used as controls. The samples were then fixed at 4 °C using 2.5% glutaraldehyde for 1 day and were subsequently rinsed thrice with 0.1 M phosphate buffer for 15 min. The samples were then fixed with 1% OsO_4_ solution for 1 h and then dehydrated in 10%, 30%, 50%, 70%, 90%, and 100% ethanol at 10 min intervals. Gold coating of the samples was then carried out after drying at the critical point, followed by SEM observations.

## 3. Result

### 3.1. Chemistry

Hybrid molecules are defined as chemical entities with two or more structural domains having different biological functions and dual activity, indicating that a hybrid molecule acts as two distinct pharmacophores. Hybrid antifungal molecules **6a_1_-a_32_**, based on the conjugation of quinazolinone to pyrazolecarbamide, were designed and synthesized. Scheme 1, Scheme 2 and Scheme 3 show the synthetic route of target compounds **6a_1_-a_32_**. First, 3-(difluoromethyl)-1-methyl-1H-pyrazole-4-carboxylic acid **1** was reacted with oxalyl chloride to obtain pyrazole-4-carbonyl chloride **2**, a light yellow, oily liquid [25]. Intermediate **3** (a white product) was obtained by reacting **2** with *o*-aminobenzyl alcohol in a saturated sodium carbonate solution. Intermediate **3** was subsequently chlorinated by dichlorosulfoxide at room temperature to yield intermediate **4**, a white solid (Scheme 1). Optimized preparative procedures for all quinazolin-4-ones **5a_1_-a_32_** were conducted following László Örfi [26]. Anthranilic acid or its substituted derivatives were mixed with formamidine acetate in ethylene glycol monomethyl ether at 95–130 °C to obtain quinazolin-4-ones (Scheme 2). The target compounds of quinazolinone-scaffold-containing pyrazole carboxamides were finally obtained by reacting intermediate **4** with different substituted quinazolin-4-ones **5a_1_-a_32_** in DMF under alkaline conditions (Scheme 3)_._ The structures of all key intermediates and target compounds were confirmed using ^1^H and ^13^C NMR and HRMS, and their spectra data are shown in the Supplementary Materials.

### 3.2. X-ray Diffraction

**Figure 4:**
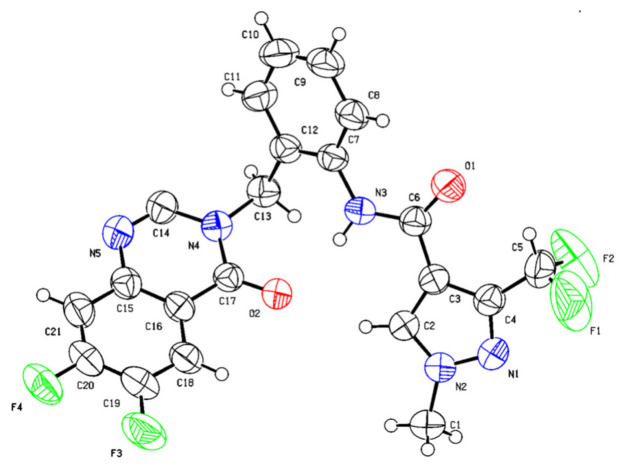
Crystal structure for **6a_11_** (CCDC number: 2218016).

To further validate the structure of the target compounds, the structure of **6a_11_** was further identified by X-ray diffraction studies (Figure 4).

### 3.3. In Vitro Antifungal Assay

Table 1 and Table 2 outline the preliminary in vitro antifungal activities of the 32 target compounds at 100 mg/L. Most of the target compounds exhibited some degree of antifungal activity, with inhibition rates ranging between 13.63% and 100% against *R. solani* (Table 1). Notably, compounds **6a_24_** and **6a_29_** showed good antifungal activity against *R. solani*, with inhibition rates of 70.26% and 70.53%, respectively. Nonetheless, these rates were lower than those of fluconazole (100%) and bixafen (100%). Hybrid antifungal molecule **6a_16_** exhibited the best antifungal activity against *R. solani*, matching that of fluconazole and bixafen.

**Figure 5:**
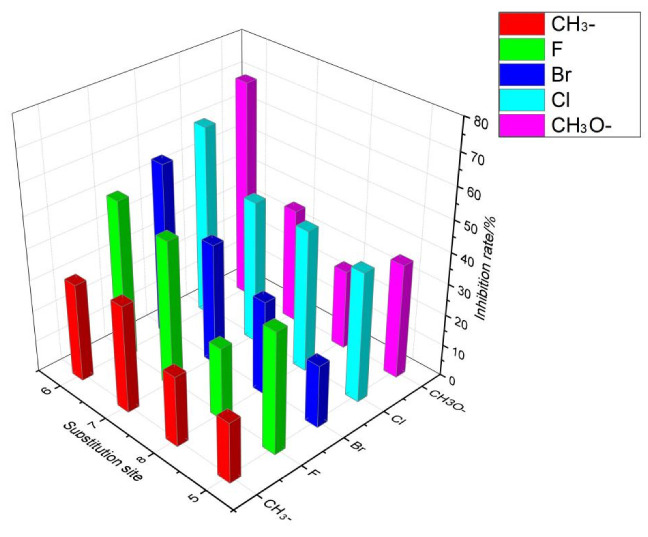
Structure–activity relationship of the target compounds.

Figure 5 shows the results of the structure–activity relationship analysis. The trend of the inhibitory activity against *R. solani* was highest when the quinazolinone of the target compounds was substituted with a bromine atom at substitution site 6. Notably, the same trend of antifungal activity was maintained when F-, Cl-, and CH_3_*O-* were substituted at different sites with a bromine atom. The fungal activity inhibition trend was 6-CH_3_O > 6-Cl > 6-Br- > 6-F > 6-CH_3_ when the substitution site was at position 6. In addition, the number and position of the substituent atoms affected the antifungal activity. For example, the structural differences of **6a_12_**, **6a_13_**, **6a_14_**, **6a_15_**, and **6a_16_** were at different positions and quantities of chlorine atoms, and their inhibition rates were 41.43%, 61.93%, 45.90%, 45.43%, and 100%, respectively. The antifungal activity at 6,8-diCl > 6-Cl > 7-Cl≈8-Cl > 5-Cl indicated that the position and number of chlorine atoms directly affected the antifungal activity.

The EC_50_ value of **6a_16_** was further tested in light of its good inhibitory characteristic. The EC_50_ of **6a_16_**, fluconazole, and bixafen were 9.06, 12.29, and 0.34 mg/L, respectively (Table 2), suggesting that the inhibitory activity of **6a_16_** against *R. solani* was comparable to that of fluconazole, but worse than that bixafen. 

### 3.4. Scanning Electron Microscopy (SEM) of the Hyphae Morphology upon Treatment of R. solani with Compound ***6a_16_***

**Figure 6:**
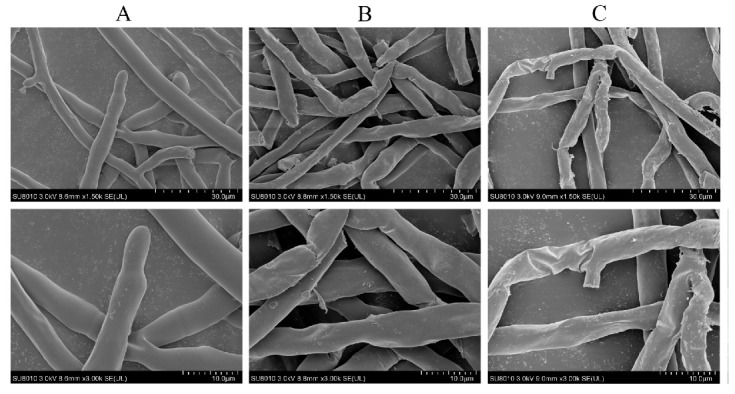
Scanning electron micrographs of *R.solani* hyphae of the control group (**A**), hyphae exposed to **6a_16_** at a concentration of 9.06 mg/L (**B**), and hyphae exposed to **6a_16_** at a concentration of 18.12 mg/L (**C**).

The morphology of fungi treated with **6a_16_** changed significantly (Figure 6). Notably, the mycelia of the control group were uniform in thickness, smooth, full on the surface, and well extended (Figure 6A). However, the mycelia appeared to fold and collapse after treatment with 9.06 mg/L and 18.12 mg/L of compound **6a_16_** (Figure 6C).

## 4. Discussion

The nucleophilic substitution of quinazolinone can result in *N*- and *O*-substitutions [31,32]. In this study, the quinazolinones **5a_1_-a_32_** were treated with chlorinated intermediate **4** in the presence of potassium hydroxide in DMF (Scheme 3). Of note, substituting quinazolin-4-ones **5a_1_-a_32_** led to the exclusive formation of *N*-substituted quinazolines, with no detection of *O*-substituted isomers. The single-crystal X-ray diffraction of compound **6a_11_** further showed that the target compound was an *N*-substituted quinazoline. Figure 4 shows the crystal structure of **6a_11_**, whose deposition number is CCDC 2218016.

Hybrid molecules are defined as chemical entities with two or more structural domains having different biological functions and dual activity, indicating that a hybrid molecule acts as two distinct pharmacophores [24]. Hybrid molecules could explore new lead compounds. Highly selective inhibitors of human α-1,3-Fucosyltransferase and acetylcholine esterase (AChE) were produced by this strategy [33,34]. In present paper, a quinazolinone structural unit and a pyrazole-containing active fragment were hybridized to create highly reactive molecules. We also obtained hybrid **6a_16_** with good antifungal activity. Therefore, molecular hybridization, based on the conjugation of quinazolinone to pyrazolecarbamide, is a useful approach for designing high antifungal candidates.

## 5. Conclusions

In this study, 32 novel pyrazolecarbamide derivatives bearing quinazolinone scaffolds were successfully designed, synthesized, and characterized in detail using ^1^H-NMR, ^13^C-NMR, and HRMS. The preliminary results of fungicidal bioassays revealed that some of the target compounds exhibited certain inhibitory activities against *R. solani*. Notably, compared with the commercial fungicide fluconazole, compound **6a_16_** exhibited excellent antifungal activities against *R. solani* by affecting the mycelial morphology. The results of this study collectively suggest that **6a_16_** is a lead compound against *R. solani* and should be further explored to enhance its utility and application.

## Figures and Tables

**Table 1:** Inhibition effect in vitro of target compounds **6a_1_-a_32_** at 100 mg/L against *R. solani*.

Compound	Inhibition Rate (%)	Compound	Inhibition Rate (%)
	*R. solani* (36 h)		*R. solani* (36 h)
**6a_1_**	54.00 ± 1.35 f	**6a_18_**	20.00 ± 0.70 qr
**6a_2_**	19.16 ± 0.95 r	**6a_19_**	56.23 ± 0.55 e
**6a_3_**	31.37 ± 1.35 n	**6a_20_**	39.13 ± 1.00 k
**6a_4_**	34.20 ± 1.00 m	**6a_21_**	30.20 ± 1.00 n
**6a_5_**	22.40 ± 1.70 p	**6a_22_**	56.33 ± 1.36 e
**6a_6_**	40.53 ± 0.67 jk	**6a_23_**	36.73 ± 0.85 l
**6a_7_**	38.56 ± 0.70 kl	**6a_24_**	70.26 ± 1.80 c
**6a_8_**	50.96 ± 0.55 g	**6a_25_**	36.93 ± 0.80 l
**6a_9_**	47.10 ± 2.23 h	**6a_26_**	25.63 ± 1.30 o
**6a_10_**	23.23 ± 1.10 p	**6a_27_**	13.63 ± 1.15 s
**6a_11_**	87.53 ± 2.20 b	**6a_28_**	21.46 ± 0.77 pq
**6a_12_**	41.43 ± 1.43 j	**6a_29_**	70.53 ± 0.87 c
**6a_13_**	61.93 ± 1.68 d	**6a_30_**	44.50 ± 1.25i
**6a_14_**	45.90 ± 2.49 hi	**6a_31_**	31.20 ± 1.57 n
**6a_15_**	45.43 ± 1.05 hi	**6a_32_**	29.70 ± 0.75 n
**6a_16_**	**100.00 ± 0.00 a**	fluconazole	**100.00 ± 0.00 a**
**6a_17_**	51.60 ± 1.44 g	bixafen	**100.00 ± 0.00 a**

Note: Data in the table are presented as means ± SD. Different lowercase letters in the same column indicate significant differences at a significance threshold of *p* < 0.05, based on Duncan’s new multiple range test.

**Table 2:** EC_50_ values of **6a_16_** against *R. solani*.

Compound	Regression Equation	EC_50_ (mg/L)	R	95% Confidence Interval (mg/L)
**6a_16_**	y = 1.3216x + 3.7350	9.06	0.9946	6.18–13.29
fluconazole	y = 1.2491x + 3.6389	12.29	0.9984	8.13–18.57
bixafen	y = 1.3358x + 5.6195	0.34	0.9959	0.23–0.50

## Data Availability

The data presented in this study are available in article and supplementary material.

## Supplementary Materials

The following supporting information can be downloaded at: https://www.mdpi.com/article/10.3390/cimb44110380/s1.
