# Supplementary material for: Design, Synthesis, and Bioactivity of Novel Quinazolinone Scaffolds Containing Pyrazole Carbamide Derivatives as Antifungal Agents
Source: Curr Issues Mol Biol. 2022 Nov 12;44(11):5605–21. doi: 10.3390/cimb44110380 (PMC9689193; doi:10.3390/cimb44110380)
Supplement: Supplementary file 1 [file cimb-44-00380-s001.zip › cimb-2012907-supplementary.pdf]

# Supplementary Materials

# Design, Synthesis, and Bioactivity of Novel Quinazolinone Scaffolds Containing Pyrazole Carbamide Derivatives as Antifungal Agents

**Zhiwei Lei [1,2,*], Jianmei Yao [2], Huifang Liu [2], Xianjin Bai [3], Xingsi Gao [3], Qiuyuan Pan [3] and Wen Yang [2]**

[1] Key Laboratory of Green Pesticide and Agricultural Bioengineering, Ministry of Education, Guizhou University, Huaxi District, Guiyang 550025, China
[2] Tea Research Institute, Guizhou Academy of Agricultural Sciences, Huaxi District, Guiyang 550006, China
[3] School of Biological Sciences, Guizhou Education University, Wudang District, Guiyang 550000, China
* Correspondence: leizhiwei816@163.com

*Instruments and Chemicals*

$^{1}H$ and $^{13}C$ NMR spectra were recorded in $CDCl_3$ or DMSO-$d_6$ using 600 and 150 MHz spectrophotometers (Bruker BioSpin GmbH, Rheinstetten, Germany), respectively. All solvents were distilled and dried using standard methods before use.

*General procedure for the preparation of quinazolin-4-ones (5a$_1$-a$_{32}$)*

Anthranilic acid or its substituted derivatives (10 mmol) were mixed with formamidine acetate (20 mmol) in ethylene glycol monomethyl ether and the reaction mixture was subsequently stirred at 95–130°C. The mixture was then poured into cold water, thereby precipitating a large amount of solid. The crude product was subsequently obtained by filtration and was dissolved in a hot 10% NaOH solution, heated for 5–6 minutes with charcoal, and filtered, and the clear solution was subsequently neutralized (pH=7) using 1N HCl. The precipitated crystals were filtered out, washed with cooled water, and dried to obtain the quinazolin-4-ones.

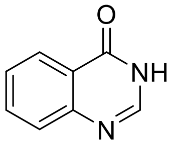


5a$_1$

quinazolin-4(3*H*)-one, white solid, yield 86%, 217.5-218.3°C. $^{1}H$ NMR (600 MHz, DMSO-$d_6$) $\delta$ 12.24 (s, 1H), 8.12 (dd, $J$= 7.9, 1.6 Hz, 1H), 8.09 (s, 1H), 7.81 (ddd, $J$= 8.5, 7.1, 1.6 Hz, 1H), 7.68–7.63 (m, 1H), 7.52 (ddd, $J$ = 8.1, 7.1, 1.2 Hz, 1H).$^{13}C$ NMR (150 MHz, DMSO-$d_6$) $\delta$ 160.73, 148.77, 145.39, 134.32, 127.24, 126.75, 125.84, 122.64.HRMS (ESI): calculated for $C_8H_6N_2O$ $[M+H]^+$:147.05529, found: 147.05556.

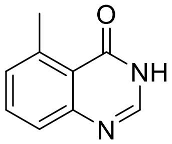


5a$_2$

5-methylquinazolin-4(3*H*)-one, white solid, yield 86%, 223.5-224.9 °C. $^{1}H$ NMR (600 MHz, DMSO-$d_6$) $\delta$ 12.01 (s, 1H), 7.99 (s, 1H), 7.60 (dd, $J$ = 7.7 Hz, 1H), 7.49-7.43(m, 1H), 7.23 (dt, $J$ = 7.4, 1.1 Hz, 1H), 2.76 (s, 3H).$^{13}C$ NMR (150 MHz, DMSO-$d_6$) $\delta$ 161.67, 150.32, 145.14, 140.03, 133.29, 128.96, 125.34, 22.55. HRMS (ESI): calculated for $C_9H_8N_2O$ $[M+H]^+$:161.07094, found: 161.07095.

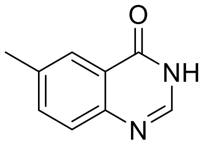

5a3

6-methylquinazolin-4(3*H*)-one,white solid, yield 85%, 259.8-259.9℃.$^{1}$H NMR (600 MHz, DMSO-$d_6$) $\delta$ 12.15 (s, 1H), 8.02 (s, 1H), 7.91 (d, *J* = 2.0 Hz, 1H), 7.62 (dd, *J* = 8.3, 2.0 Hz, 1H), 7.56 (d, *J* = 8.3 Hz, 1H), 2.43 (s, 3H). $^{13}$C NMR (150 MHz, DMSO-$d_6$) $\delta$ 160.73, 146.79, 144.58, 136.51, 135.65, 127.14, 125.24, 122.40, 20.85. HRMS (ESI): calculated for $C_9H_8N_2O$ $[M+H]^+$:161.07094, found: 161.07095.

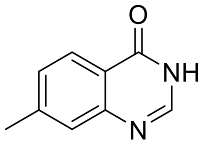

5a4

7-methylquinazolin-4(3*H*)-one, white solid, yield 81%, 243.1-244.5℃. $^1$H NMR (600 MHz, DMSO-$d_6$) $\delta$ 12.14 (s, 1H), 8.05 (s, 1H), 8.00 (d, *J* = 8.0 Hz, 1H), 7.46 (s, 1H), 7.33 (dd, *J* = 8.0, 1.6 Hz, 1H), 2.44 (s, 3H).$^{13}$C NMR (150 MHz, DMSO-d6) $\delta$ 160.63, 148.90, 145.43, 144.81, 128.15, 126.88, 125.70, 120.24, 21.32. HRMS (ESI): calculated for $C_9H_8N_2O$ $[M+H]^+$:161.07094, found: 161.07095.

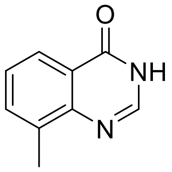

5a5

8-methylquinazolin-4(3*H*)-one, white solid, yield 82%, 255.8-256.9℃. $^1$H NMR (600 MHz, DMSO-$d_6$) $\delta$ 12.22 (s, 1H), 8.10 (s, 1H), 7.95 (dd, *J* = 8.0, 1.6 Hz, 1H), 7.65 (d, *J* = 7.2 Hz, 1H), 7.38 (t, *J* = 7.6 Hz, 1H), 2.51 (s, 3H). $^{13}$C NMR (150 MHz, DMSO-$d_6$) $\delta$ 161.16, 147.30, 144.45, 135.47, 134.82, 126.33, 123.59, 122.63, 17.36. HRMS (ESI): calculated for $C_9H_8N_2O$ $[M+H]^+$:161.07094, found: 161.07095.

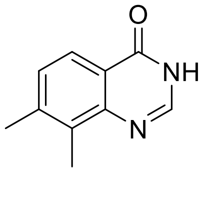

5a6

7,8-dimethylquinazolin-4(3*H*)-one,White solid, yield 83%, 261.0.8-262.8℃.$^1$H NMR (600 MHz, DMSO-$d_6$) $\delta$ 12.10 (s, 1H), 8.06 (d, *J* = 2.5 Hz, 1H), 7.84 (d, *J* = 8.1 Hz, 1H), 7.29 (d, *J* = 8.1 Hz, 1H), 2.44 (s, 3H), 2.36 (s, 3H).$^{13}$C NMR (150 MHz, DMSO-$d_6$) $\delta$ 161.19, 146.97, 144.18, 142.93, 133.43, 128.45, 122.74, 120.54, 20.56, 13.01.HRMS (ESI): calculated for $C_{10}H_{10}N_2O$ $[M+H]^+$:175.08659, found: 175.08668.

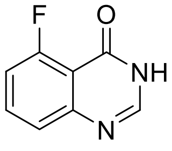

5a7

5-fluoroquinazolin-4(3*H*)-one,white solid, yield 77%, 291.7-292.6℃.$^1$H NMR (600 MHz, DMSO-$d_6$) $\delta$ 12.27 (s, 1H), 8.07 (s, 1H), 7.76 (td, *J* = 8.2, 5.5 Hz, 1H), 7.45 (d, *J* = 8.2 Hz, 1H), 7.24 (dd, *J* = 11.1, 8.2, 1H).$^{13}$C NMR (150 MHz, DMSO-$d_6$) $\delta$ 160.41 (d, *J* = 263.0 Hz), 158.02, 150.95, 146.41, 134.90 (d, *J*= 10.3 Hz), 123.31, 113.11 (d, *J* = 20.0 Hz), 112.12 (d, *J* = 6.0 Hz). HRMS (ESI): calculated for $C_8H_5FN_2O$ $[M+H]^+$: 165.04587, found: 165.04592.

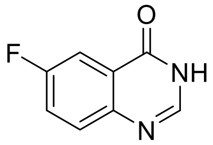

5a8

6-fluoroquinazolin-4(3*H*)-one,white solid, yield 78%, 253.4-258.6°C.$^{1}$H NMR (600 MHz, DMSO-*d*6) *δ* 12.36 (s, 1H), 8.07 (s, 1H), 7.76 (dd, *J* = 8.6, 3.0 Hz, 1H), 7.73 (dd, *J* = 8.9, 5.0 Hz, 1H), 7.67 (td, *J* = 8.6, 3.0 Hz, 1H).$^{13}$C NMR (150 MHz, DMSO-*d*6) *δ* 160.11 (ds, *J* =245.6 Hz), 160.25,145.69, 144.90,130.16 (d, *J* = 8.4 Hz), 123.92 (d, *J* =8.5 Hz), 122.85 (d, *J* = 24.2 Hz), 110.53 (d, *J* = 23.5 Hz). HRMS (ESI): calculated for $C_8H_5FN_2O$ $[M+H]^+$: 165.04587, found: 165.04592.

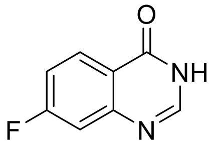

5a9

7-fluoroquinazolin-4(3*H*)-one,white solid, yield 81%, 246.2-247.2°C.$^{1}$H NMR (600 MHz, DMSO-*d*6) *δ* 12.34 (s, 1H), 8.17 (dd, *J* = 8.8, 6.3 Hz, 1H), 8.13 (s, 1H), 7.44 (dd, *J* = 10.2, 2.6 Hz, 1H), 7.38 (td, *J* = 8.8, 2.6 Hz, 1H).$^{13}$C NMR (150 MHz, DMSO-d6) *δ* 165.57 (d, *J* = 250.6 Hz), 160.05, 150.96 (d, *J* = 12.5 Hz), 146.86, 129.00 (d, *J* = 11.1 Hz), 119.65, 115.31 (d, *J* = 23.6 Hz), 112.34 (d, *J* = 21.5 Hz). HRMS (ESI): calculated for $C_8H_5FN_2O$ $[M+H]^+$:165.04587, found: 165.04592.

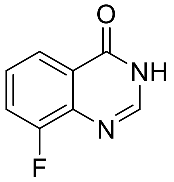

5a10

8-fluoroquinazolin-4(3*H*)-one, white solid, yield 75%, 274.5-274.6°C. $^{1}$H NMR (600 MHz, DMSO-*d*6) *δ* 12.45 (s, 1H), 8.14 (s, 1H), 7.94 – 7.90 (m, 1H), 7.67 (ddd, *J* = 10.7, 8.0, 1.3 Hz, 1H), 7.50 (td, *J* = 8.0, 4.8 Hz, 1H).$^{13}$C NMR (150 MHz, DMSO-d6) *δ* 159.85 (d, *J* = 3.5 Hz), 156.48 (d, *J* = 253.4 Hz), 146.03, 137.96 (d, *J* = 11.3 Hz), 127.01 (d, *J* = 7.7 Hz), 124.67, 121.60 (d, *J* = 3.7 Hz), 119.77 (d, *J* = 18.6 Hz).HRMS (ESI): calculated for $C_8H_5FN_2O$ $[M+H]^+$:165.04587, found: 165.04592.

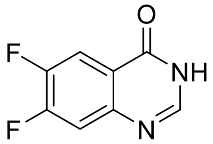

5a11

6,7-difluoroquinazolin-4(3*H*)-one, white solid, yield 83%, 259.3-260.9°C.$^{1}$H NMR (600 MHz, DMSO-*d*6) *δ* 12.47 (s, 1H), 8.13 (s, 1H), 8.00 (dd, *J* = 10.4, 8.7 Hz, 1H), 7.72 (dd, *J* = 11.4, 7.3 Hz, 1H). $^{13}$C NMR (150 MHz, DMSO-*d*6) *δ* 159.50, 153.75(dd, *J* = 253.7, 13.6 Hz), 148.54 (dd, *J* = 249.1, 13.6 Hz), 146.82 (d, *J* = 11.1 Hz), 146.36, 119.93 (d, *J* = 6.1 Hz), 115.27 (d, *J* = 17.4 Hz), 113.39 (d, *J* = 18.7 Hz). HRMS (ESI): calculated for $C_8H_4F_2N_2O$ $[M+H]^+$:183.03645, found: 183.03604.

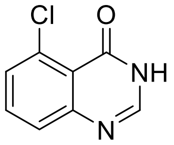

5a12

5-chloroquinazolin-4(3*H*)-one,white solid, yield 68%, 207.7-209.0°C. $^{1}$H NMR (600 MHz, DMSO-$d_6$) $\delta$ 12.29 (s, 1H), 8.07 (s, 1H), 7.72 (t, *J* = 8.0 Hz, 1H), 7.60 (dd, *J* = 8.2, 1.2 Hz, 1H), 7.52 (dd, *J* = 7.8, 1.2 Hz, 1H). $^{13}$C NMR (150 MHz, DMSO-$d_6$) $\delta$ 159.00, 151.31, 146.28, 134.24, 132.42, 129.25, 126.89, 119.63. HRMS (ESI): calculated for $C_8H_5ClN_2O$ $[M+H]^+$:181.01632, found: 181.01650.

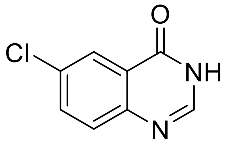


5a13

6-chloroquinazolin-4(3*H*)-one, white solid, yield 71%, 269.8-271.1°C.$^{1}$H NMR (600 MHz, DMSO-$d_6$) $\delta$ 12.42 (s, 1H), 8.10 (s, 1H), 8.01 (d, *J* = 2.5 Hz, 1H), 7.80 (dd, *J* = 8.7, 2.5 Hz, 1H), 7.66 (d, *J* = 8.7 Hz, 1H). $^{13}$C NMR (150 MHz, DMSO-$d_6$) $\delta$ 159.86, 147.53, 145.94, 134.50, 131.13, 129.57, 124.89, 123.92. HRMS (ESI): calculated for $C_8H_5ClN_2O$ $[M+H]^+$: 181.01632, found: 181.01650.

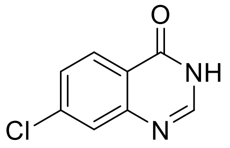


5a14

7-chloroquinazolin-4(3*H*)-one, white solid, yield 69%, 251.0-253.0°C.$^{1}$H NMR (600 MHz, DMSO-$d_6$) $\delta$ 12.36 (s, 1H), 8.11 (s, 1H), 8.06 (d, *J* = 8.5 Hz, 1H), 7.64 (d, *J* = 2.1 Hz, 1H), 7.48 (dd, *J* = 8.5, 2.1 Hz, 1H). $^{13}$C NMR (150 MHz, DMSO-$d_6$) $\delta$ 160.24, 149.87, 146.92, 138.98, 127.97, 127.04, 126.40, 121.47. HRMS (ESI): calculated for $C_8H_5ClN_2O$ $[M+H]^+$: 181.01632, found: 181.01650.

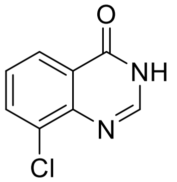


5a15

8-chloroquinazolin-4(3*H*)-one, white solid, yield 71%, 305.5-307.2°C.$^{1}$H NMR (600 MHz, DMSO-$d_6$) $\delta$ 12.50 (s, 1H), 8.21 (s, 1H), 8.07 (dd, *J* = 8.0, 1.4 Hz, 1H), 7.95 (dd, *J* = 7.8, 1.4 Hz, 1H), 7.48 (t, *J* = 7.8 Hz, 1H). $^{13}$C NMR (150 MHz, DMSO-$d_6$) $\delta$ 160.24, 146.38, 145.23, 134.46, 130.82, 127.10, 125.03, 124.36. HRMS (ESI): calculated for $C_8H_5ClN_2O$ $[M+H]^+$: 181.01632, found: 181.01650.

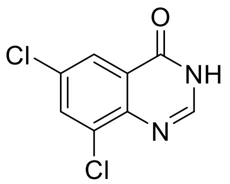


5a16

6,8-chloroquinazolin-4(3*H*)-one, white solid, yield 68%, >310 °C.$^{1}$H NMR (600 MHz, DMSO-$d_6$) $\delta$ 12.68 (s, 1H), 8.24 (s, 1H), 8.12 (s, 1H), 8.02 (s, 1H). $^{13}$C NMR (150 MHz, DMSO-$d_6$) $\delta$ 159.32, 146.81, 144.27, 134.04, 132.38, 130.72, 125.05, 124.18. HRMS (ESI): calculated for $C_8H_4Cl_2N_2O$ $[M+H]^+$: 214.97734, found: 214.97717.

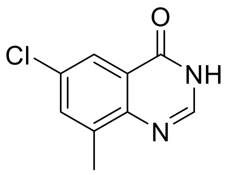

5a17

**6. -chloro-8-methylquinazolin-4(3*H*)-one,white solid, yield 59%, >310 °C.$^1$H NMR (600 MHz, DMSO-*d*$_6$) δ 12.42 (s, 1H), 8.13 (s, 1H), 7.88 (d, *J* = 2.5 Hz, 1H), 7.73 (d, *J* = 2.5 Hz, 1H), 2.51 (s, 3H).$^{13}$C NMR (150 MHz, DMSO-*d*$_6$) δ 160.16, 146.16, 144.99, 138.58, 134.47, 130.54, 123.79, 122.40, 17.08. HRMS (ESI): calculated for $C_9H_7ClN_2O$ [M+H]$^+$: 195.03197, found: 195.03183.**

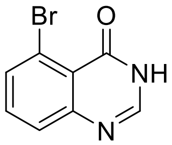

5a18

5-bromoquinazolin-4(3*H*)-one, white solid, yield 68%, 243.6-243.7°C.$^1$H NMR (600 MHz, DMSO-*d*$_6$) δ 12.31 (s, 1H), 8.08 (s, 1H), 7.72 (dd, *J* = 6.9, 2.1 Hz, 1H), 7.66 -7.58 (m, 2H).$^{13}$C NMR (150 MHz, DMSO-*d*$_6$) δ 159.01, 151.12, 145.96, 134.44, 132.91, 127.51, 120.49, 119.91. HRMS (ESI): calculated for $C_8H_5BrN_2O$ [M+H]$^+$: 224.96580, found: 224.96560.

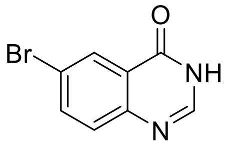

5a19

6-bromoquinazolin-4(3*H*)-one, white solid, yield 41%, 270.6-271.9 °C.$^1$H NMR (600 MHz, DMSO-*d*$_6$) δ 12.42 (s, 1H), 8.16 (d, *J* = 2.4 Hz, 1H), 8.12 (s, 1H), 7.93 (dd, *J* = 8.7, 2.4 Hz, 1H), 7.60 (d, *J* = 8.7 Hz, 1H). $^{13}$C NMR (150 MHz, DMSO-*d*$_6$) δ 159.75, 147.74, 146.10, 137.24, 129.66, 128.02, 127.93, 124.26, 119.30. HRMS (ESI): calculated for $C_8H_5BrN_2O$ [M+H]$^+$: 224.96580, found: 224.96560.

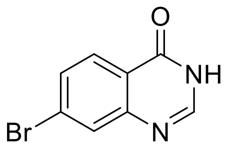

5a20

7-bromoquinazolin-4(3*H*)-one, white solid, yield 57%, 270.6-271.9 °C.$^1$H NMR (600 MHz, DMSO-*d*$_6$) δ 12.38 (s, 1H), 8.11 (s, 1H), 8.00 (d, *J* = 8.5 Hz, 1H), 7.83 (d, *J* = 2.0 Hz, 1H), 7.65 (dd, *J* = 8.5, 2.0 Hz, 1H). $^{13}$C NMR (150 MHz, DMSO-*d*$_6$) δ 160.42, 149.94, 146.92, 129.88, 129.50, 128.04, 127.99, 121.78. HRMS (ESI): calculated for $C_8H_5BrN_2O$ [M+H]$^+$: 224.96580, found: 224.96560.

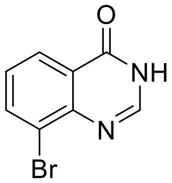

5a21

8-bromoquinazolin-4(3*H*)-one, white solid, yield 63%, >310 °C. $^1$H NMR (600 MHz, DMSO-*d*$_6$) δ 12.50 (s, 1H), 8.21 (s, 1H), 8.12 (td, *J* = 7.5, 1.4 Hz, 2H), 7.42 (t, *J* = 7.8 Hz, 1H).$^{13}$C NMR (150 MHz, DMSO-*d*$_6$) δ 160.25, 146.46, 146.24, 137.78, 127.61, 125.73, 124.27, 121.84. HRMS (ESI): calculated for $C_8H_5BrN_2O$ [M+H]$^+$: 224.96580, found: 224.96560.

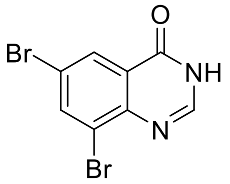

5a22

6,8-dibromoquinazolin-4(3*H*)-one, white solid, yield 48%, >310 °C. $^{1}$H NMR (600 MHz, DMSO-*d*$_6$) *δ* 12.67 (s, 1H), 8.35 (s, 1H), 8.24 (s, 1H), 8.18 (s, 1H). $^{13}$C NMR (150 MHz, DMSO-*d*$_6$) *δ* 159.33, 147.02, 145.60, 139.65, 127.95, 125.26, 123.44, 119.11. HRMS (ESI): calculated for $C_8H_4Br_2N_2O$ [M+H]$^+$: 302.87632, found: 302.87534.

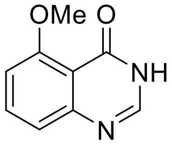

5a23

5-methoxyquinazolin-4(3*H*)-one, white solid, yield 72%, 210.5 - 211.2 °C. $^{1}$H NMR (600 MHz, DMSO-*d*$_6$) *δ* 11.91 (s, 1H), 7.95 (s, 1H), 7.66 (t-like, *J* = 8.2 Hz, 1H), 7.16 (d, *J* = 8.0 Hz, 1H), 6.99 (d, *J* = 8.2 Hz, 1H) , 3.84 (s, 3H).$^{13}$C NMR (150 MHz, DMSO-*d*$_6$) *δ* 159.77, 159.04, 151.35, 145.83, 134.75, 119.12, 112.34, 108.46, 56.06. HRMS (ESI): calculated for $C_8H_4$Br2$N_2O$ [M+H]$^+$: 302.87632, found: 302.87534.

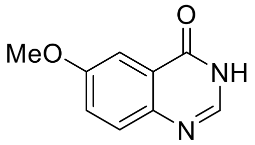

5a24

6-methoxyquinazolin-4(3*H*)-one, white solid, yield 70%, 246.6-247.9 °C.$^{1}$H NMR (600 MHz, DMSO-*d*$_6$) *δ* 12.18 (s, 1H), 7.98 (s, 1H), 7.62 (d, *J* = 8.9 Hz, 1H), 7.50 (d, *J* = 3.0 Hz, 1H), 7.41 (dd, *J* = 8.9, 3.0 Hz, 1H), 3.87 (s, 3H).$^{13}$C NMR (150 MHz, DMSO-*d*$_6$) *δ* 160.52, 157.74, 143.21, 143.10, 128.94, 123.75, 123.45, 105.82, 55.61. HRMS (ESI): calculated for $C_8H_4$Br2$N_2O$ [M+H]$^+$: 302.87632, found: 302.87534.

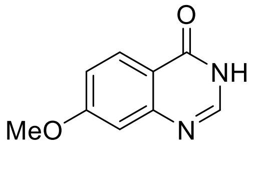

5a25

7-methoxyquinazolin-4(3*H*)-one, white solid, yield 67%, 263.8-263.9 °C. $^{1}$H NMR (600 MHz, DMSO-*d*$_6$) *δ* 12.09 (s, 1H), 8.05 (s, 1H), 8.01 (dd, *J* = 8.2, 1.0 Hz, 1H), 7.11 – 7.06 (m, 2H), 3.88 (s, 3H). $^{13}$C NMR (150 MHz, DMSO-*d*$_6$) *δ* 163.92, 160.29, 151.02, 146.00, 127.46, 116.19, 116.03, 108.40, 55.69. HRMS (ESI): calculated for $C_8H_4$Br2$N_2O$ [M+H]$^+$: 302.87632, found: 302.87534.

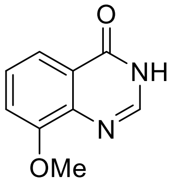

5a26

8-methoxyquinazolin-4(3*H*)-one, white solid, yield 89%, 296.6 - 297.9 °C. $^{1}$H NMR (600 MHz, DMSO-*d*$_6$) *δ* 12.25 (s, 1H), 8.02 (s, 1H), 7.66 (dd, *J* = 8.0, 1.3 Hz, 1H), 7.44 (dd, *J* = 8.0 Hz, 1H), 7.36 (dd, *J* = 8.0, 1.3 Hz, 1H), 3.89 (s, 3H).$^{13}$C NMR (150 MHz, DMSO-*d*$_6$) *δ* 160.61, 154.54, 143.94, 139.27, 127.03, 123.68, 116.78, 114.93, 55.96. HRMS (ESI): calculated for $C_8H_4$Br2$N_2O$ [M+H]$^+$: 302.87632, found: 302.87534.

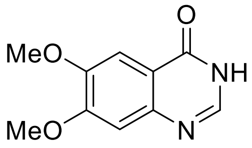

5a27

7,8-dimethoxyquinazolin-4(3*H*)-one, white solid, yield 80%, 264.3.6-268.3 °C. $^{1}H$ NMR (600 MHz, DMSO-$d_6$) $\delta$ 12.07 (s, 1H), 7.98 (s, 1H), 7.43 (s, 1H), 7.12 (s, 1H), 3.89 (s, 3H), 3.86 (s, 3H).$^{13}C$ NMR (150 MHz, DMSO-$d_6$) $\delta$ 160.15, 154.52, 148.62, 144.92, 143.90, 115.64, 108.05, 104.96, 55.99, 55.75. HRMS(ESI): calculated for $C_{10}H_{10}N_2O_3$ [M+H]$^+$: 207.07642, found: 207.07626.

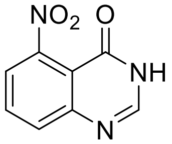

5a28

5-nitroquinazolin-4(3*H*)-one, red brown solid, yield 39%, 253.2-255.1 °C. $^{1}H$ NMR (600 MHz, DMSO-$d_6$) $\delta$ 12.74 (s, 1H), 8.23 (s, 1H), 7.98-7.93 (m, 1H), 7.88 (dd, *J* = 8.3, 1.2 Hz, 1H), 7.81 (dd, *J* = 7.6, 1.2 Hz, 1H).$^{13}C$ NMR (150 MHz, DMSO-$d_6$) $\delta$ 157.43, 149.70, 148.18, 147.22, 134.66, 130.06, 120.50, 113.06. HRMS(ESI): calculated for $C_8H_5N_3O_3$ [M+H]$^+$: 192.04037, found: 192.04003.

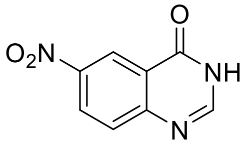

5a29

6-nitroquinazolin-4(3*H*)-one, light yellow solid, yield 42%, 283.6-284.2 °C. $^{1}H$ NMR (600 MHz, DMSO-$d_6$) $\delta$ 12.73 (s, 1H), 8.75 (d, *J* = 2.7 Hz, 1H), 8.51 (dd, *J* = 8.9, 2.7 Hz, 1H), 8.28 (s, 1H), 7.82 (d, *J* = 8.9 Hz, 1H). $^{13}C$ NMR (150 MHz, DMSO-$d_6$) $\delta$ 160.11, 153.00, 148.91, 145.02, 129.15, 128.32, 122.73, 121.96. HRMS(ESI): calculated for $C_8H_5N_3O_3$ [M+H]$^+$: 192.04037, found: 192.04003.

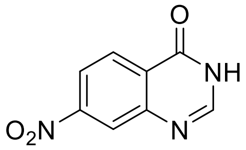

5a30

7-nitroquinazolin-4(3*H*)-one, red brown solid, yield 71%, 266.9-267.3 °C. $^{1}H$ NMR (600 MHz, DMSO-$d_6$) $\delta$ 12.66 (s, 1H), 8.35 (d, *J* = 2.3 Hz, 1H), 8.32 (d, *J* = 8.7 Hz, 1H), 8.25 (s, 1H), 8.22 (dd, *J* = 8.7, 2.3 Hz, 1H). $^{13}C$ NMR (150 MHz, DMSO-$d_6$) $\delta$ 159.83, 151.04, 149.14, 147.73, 128.20, 126.98, 122.21, 120.35. HRMS(ESI): calculated for $C_8H_5N_3O_3$ [M+H]$^+$: 192.04037, found: 192.04003.

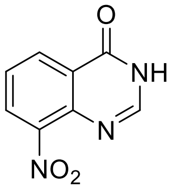

5a31

8-nitroquinazolin-4(3*H*)-one, red brown solid, yield 49%, 216.9-217.3 °C. $^{1}H$ NMR (600 MHz, DMSO-$d_6$) $\delta$ 12.70 (s, 1H), 8.34 (dd, *J* = 8.0, 1.5 Hz, 1H), 8.27 (dd, *J* = 7.8, 1.5 Hz, 1H), 8.24 (s, 1H), 7.65 (t-like, *J* = 8.0 Hz, 1H).$^{13}C$ NMR (150 MHz, DMSO-$d_6$) $\delta$ 159.33, 148.16, 146.66, 140.47, 129.56, 127.74, 126.42, 124.04. HRMS(ESI): calculated for $C_8H_5N_3O_3$ [M+H]$^+$: 192.04037, found: 192.04003.

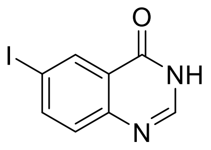

5a32

6-iodoquinazolin-4(3*H*)-one, white solid, yield 88%, 271.7-273.2 °C. $^{1}H$ NMR (600 MHz, DMSO-$d_6$) $\delta$ 12.40 (s, 1H), 8.37 (d, *J* = 2.1 Hz, 1H), 8.12 (s, 1H), 8.08 (dd, *J* = 8.5, 2.1 Hz, 1H), 7.45 (d, *J* = 8.5 Hz, 1H).$^{13}C$ NMR (150 MHz, DMSO-$d_6$) $\delta$ 159.53, 148.03, 146.15, 142.74, 134.19, 129.45, 124.46, 91.86. HRMS(ESI): calculated for $C_8H_5IN_2O$ $[M+H]^+$: 272.95193, found: 272.95200.
